# Crystal structures of five 1-alkyl-4-aryl-1,2,4-triazol-1-ium halide salts

**DOI:** 10.1107/S2056989015009019

**Published:** 2015-05-16

**Authors:** Marites A. Guino-o, Meghan O. Talbot, Michael M. Slitts, Theresa N. Pham, Maya C. Audi, Daron E. Janzen

**Affiliations:** a Chemistry Department, University of St Thomas, Mail OSS 402, Summit Avenue, St Paul, MN 55105-1079, USA; b Dept of Chemistry and Biochemistry, St. Catherine University, 2004 Randolph Avenue, St Paul, MN 55105, USA

**Keywords:** crystal structure, 1,2,4-triazolium salts, C-H⋯halide inter­actions

## Abstract

The asymmetric units for the salts 4-(4-fluoro­phen­yl)-1-isopropyl-1,2,4-triazol-1-ium iodide, C_11_H_13_FN_3_^+^·I^−^, (**1**), 1-isopropyl-4-(4-methyl­phen­yl)-1,2,4-triazol-1-ium iodide, C_12_H_16_N_3_^+^·I^−^, (**2**), 1-isopropyl-4-phenyl-1,2,4-triazol-1-ium iodide, C_11_H_14_N_3_^+^·I^−^, (**3**), and 1-methyl-4-phenyl-1,2,4-triazol-1-ium iodide, C_9_H_10_N_3_^+^·I^−^, (**4**), contain one cation and one iodide ion, whereas in 1-benzyl-4-phenyl-1,2,4-triazol-1-ium bromide monohydrate, C_15_H_14_N_3_^+^·Br^−^·H_2_O, (**5**), there is an additional single water mol­ecule. There is a predominant C—H⋯*X*(halide) inter­action for all salts, resulting in a two-dimensional extended sheet network between the triazolium cation and the halide ions. For salts with *para*-substitution on the aryl ring, there is an additional π–anion inter­action between a triazolium carbon and iodide displayed by the layers. For salts without the *para*-substitution on the aryl ring, the π–π inter­actions are between the triazolium and aryl rings. The melting points of these salts agree with the predicted substituent inductive effects.

## Abstract 2

To investigate the predominant inter­molecular inter­actions in 1-alkyl-4-aryl-1,2,4-triazol-1-ium halide salts, five salts were prepared and crystallographically characterized. The halide ions generally inter­act with the H atoms of the triazolium cation forming extended sheets. When the aryl ring lies on the plane of the triazolium cation, the cationic core formed two-dimensional networks that lead into layer-like assembled structures. The triazolium core exhibits π–π inter­actions with the iodide and/or the aryl ring of another layer. The melting-point temperatures of each salt were also determined.

## Chemical context   

Literature syntheses of asymmetric 1,2,4-triazolium cations have increased in recent years due to their utility as cations in ionic liquids (ILs) and as precursors to *N-*heterocyclic carbenes (NHCs) (Dwivedi *et al.*, 2014 ▸; Meyer & Strassner, 2011 ▸; Mochida *et al.*, 2011 ▸; Nelson, 2015 ▸; Porcar *et al.*, 2013 ▸; Strassner *et al.*, 2013 ▸). Most structural analyses of these cations have been performed to understand how the inter­molecular features of ILs affect their physical properties. (Porcar *et al.*, 2013 ▸).
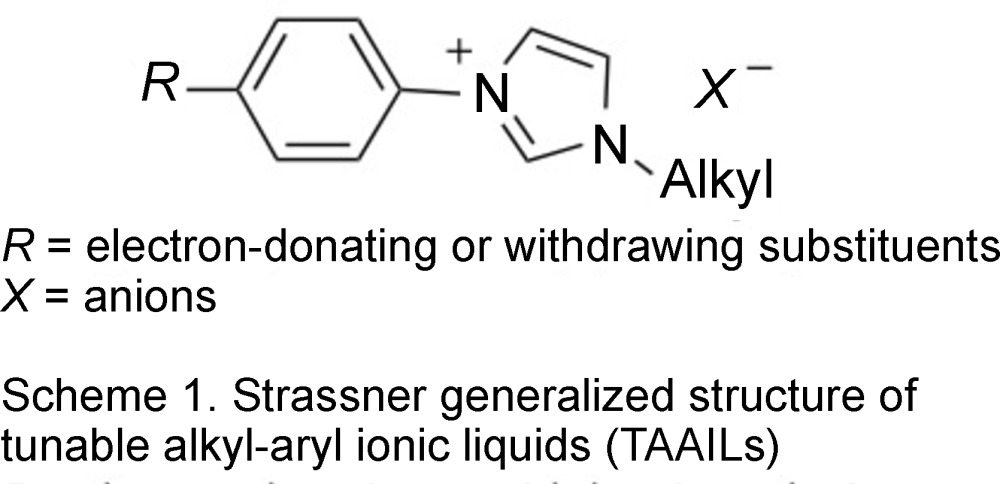


Most recently, Strassner has introduced a new group of ionic liquids called ‘TAAILs’ (tunable ar­yl–alkyl ionic liquids) (Ahrens *et al.*, 2009 ▸). The idea is to tune the properties of the ionic liquids through modification of the aryl and alkyl substituents of an imidazole cation (Scheme 1). The new cations can still be combined with the previously used anions in ILs. These workers have demonstrated that electron-donating *para*-substituents on the aryl group lower the melting point, while electron-withdrawing *para*-substituents raise the melting point. Thus one can tune the IL properties through the introduction of an electronic variation through *para*-substitution on the aryl rings. This group has also extended the concept to the 1,2,4-triazolium cation core (Meyer & Strassner, 2011 ▸).

Our group became inter­ested in learning how the ar­yl/alkyl substituents on the triazole ring affect the solid-state structures of the salts because strategic choice of substituents should allow tailorable π–π inter­actions as predicted by Strassner’s group (Meyer & Strassner, 2011 ▸). Herein, the preparation and crystal structure analyses of salts (**1**)–(**5**) are discussed (Scheme 2). Cations (**1**)–(**3**) compare the inductive effects of the electronic *para*-substituents in the aryl group, while cations (**3**)–(**5**) contrast the steric bulk of the alkyl substituents. None of the compounds presented here are ILs because we used iodide or bromide counter-anions to facilitate crystal formation. Understanding inter­actions in the solid state may help better design systems where the triazolium cations are needed.
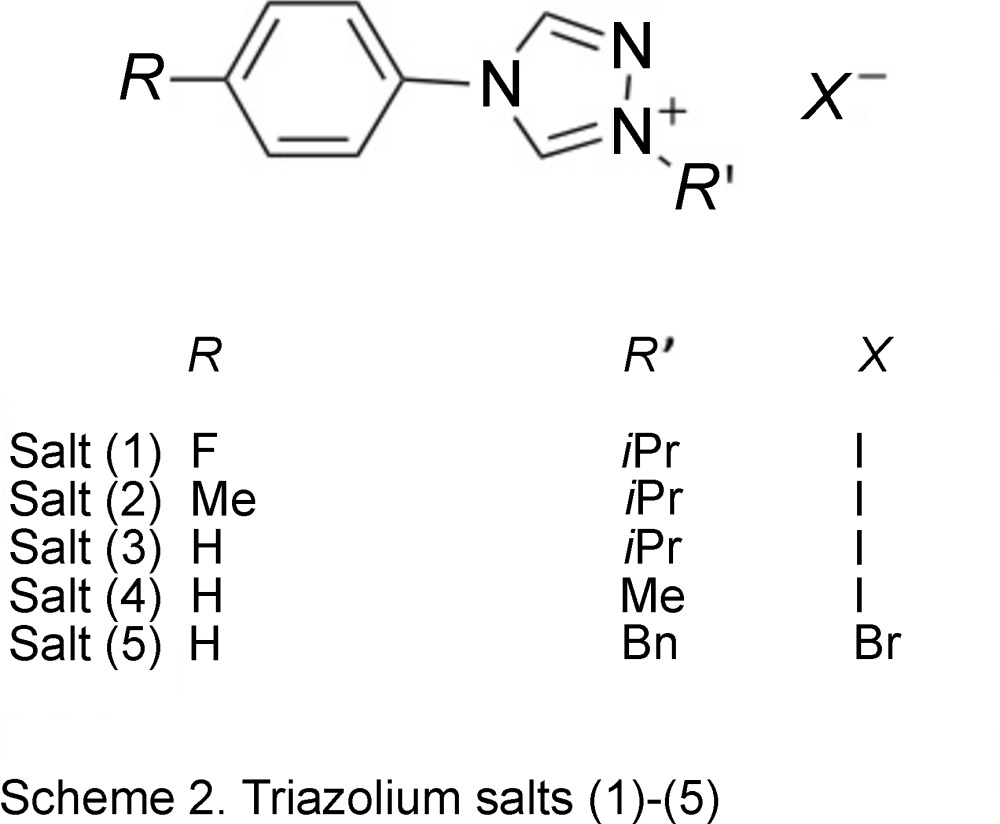


## Structural commentary   

Salts (**1**) and (**2**) crystallized in the ortho­rhom­bic space group *Pccn*, salt (**3**) in the monoclinic space group *P*2_1_ */n*, and salt (**5**) in the monoclinic space group *C*2/*c*. Salt (**4**) crystallized in the non-centrosymmetric space group *Cc* with a Flack parameter of −0.01 (2) indicating the absolute structure is well determined.

The asymmetric unit for all salts contains one cation and one iodide or bromide ion, except for salt (**5**), where there is an additional single water mol­ecule. The bond lengths in the triazolium rings for all salts indicate aromaticity with C—N and N—N bond distances in the narrow range of 1.292 (6)–1.365 (5) Å for (**1**), 1.304 (5)–1.365 (4) Å for (**2**), 1.301 (3)–1.374 (3) Å for (**3**), 1.297 (6)–1.370 (5) Å for (**4**), and 1.299 (4)–1.375 (4) Å for (**5**); with N—C—N bond angles of 107.7 (4)° for (**1**), 107.5 (3)° for (**2**), 107.3 (2)° for (**3**), 107.5 (3)° for (**4**), and 107.2 (2)° for (**5**). These values are very similar to those reported for 4-phenyl-1-ethyl-4*H*-1,2,4-triazolium bromide, in which the C—N and N—N bond distances range is 1.301 (3)–1.469 (4) Å and the N—C—N bond angle is 107.8 (2)° (Meyer & Strassner, 2011 ▸). The phenyl ring for these salts lies in almost the same plane as the triazole ring with torsion angles of 6.5 (7)° for (**1**), 24.1 (5)° for (**2**), 12.9 (4)° for (**4**), and 3.1 (4)° for (**5**); except for salt (**3**) where the phenyl ring is almost perpendicular to the triazole ring with a torsion angle of 65.1 (3)°. The torsion angle between the phenyl and triazole rings for the reported triazolium bromide is 5.8 (4)° (Meyer & Strassner, 2011 ▸). There are no significant intra­molecular inter­actions found in any of the salts.

## Supra­molecular features   

**Figure 1:**
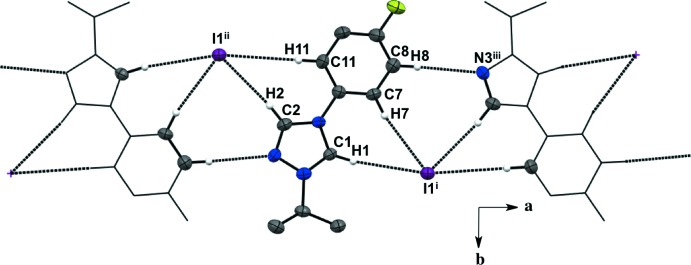
Extended sheet network viewed along the *c* axis of salt (**1**). H atoms not participating in the extended sheet network are not shown. For symmetry codes, see Table 1 ▸.

**Figure 2:**
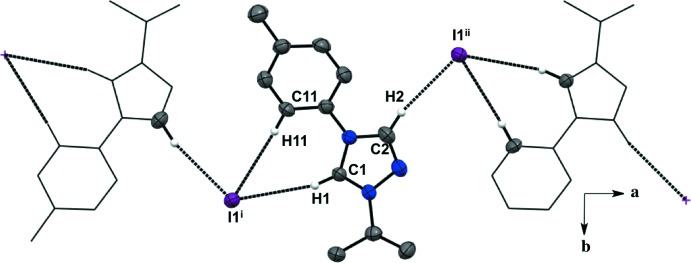
Extended sheet network viewed along the *c* axis of salt (**2**). H atoms not participating in the extended sheet network are not shown. For symmetry codes, see Table 2 ▸.

**Figure 3:**
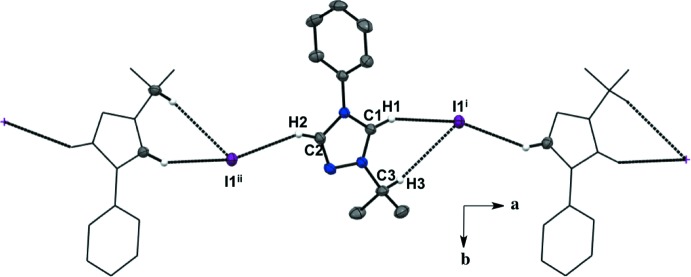
Extended sheet network viewed along the *c* axis of salt (**3**). H atoms not participating in the extended sheet network are not shown. For symmetry codes, see Table 3 ▸.

**Figure 4:**
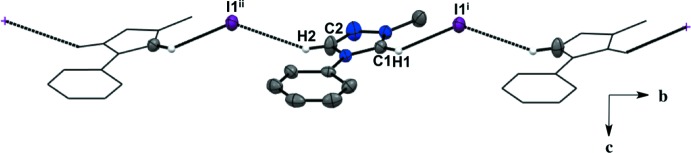
Extended sheet network viewed along the *a* axis of salt (**4**). H atoms not participating in the extended sheet network are not shown. For symmetry codes, see Table 4 ▸.

**Figure 5:**
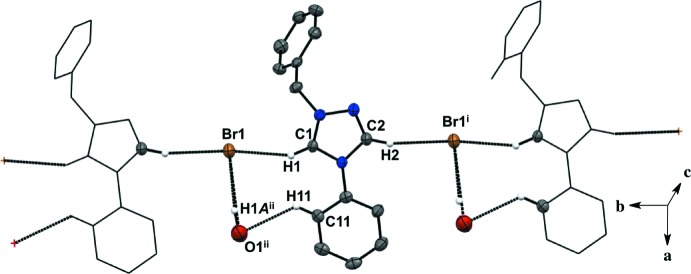
Extended sheet network of salt (**5**). H atoms not participating in the extended sheet network are not shown. For symmetry codes, see Table 5 ▸.

**Figure 6:**
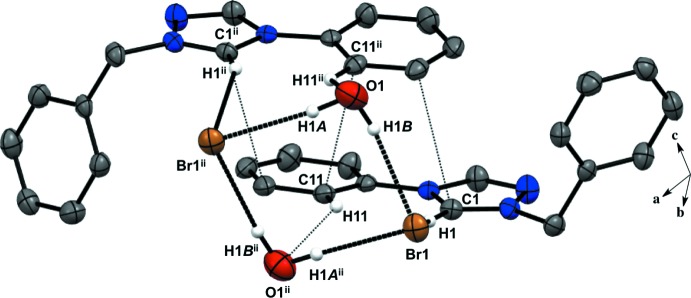
Donor–acceptor inter­actions of bromide ions and water mol­ecules with each other, with the triazolium C—H, and the *ortho* C—H of the aryl ring found in salt (**5**). H atoms not participating in the inter­actions are not shown. For symmetry codes, see Table 5 ▸.

For all five salts, there is a predominant C—H⋯halide inter­molecular inter­action between the hydrogen atoms in the triazolium ring and the counter ions, forming an extended network (Figs. 1–5 ▸ ▸ ▸ ▸ ▸ and Tables 1 ▸–5 ▸ ▸ ▸ ▸). For the asymmetric unit in salt (**1**), there are a total of four C—H⋯I^−^ inter­molecular inter­actions with two neighboring mol­ecules (Fig. 1 ▸, Table 1 ▸). Each iodide ion inter­acts with two C—H moieties from the triazolium ring and two from the *ortho* C—H moieties of the aryl group. There is an additional C—H⋯N inter­action between the *meta* C—H of the aryl ring and the triazolium nitro­gen atom. The fluorine substituent in the *para-* position of the aryl ring is not an acceptor in any of the C—H inter­actions in salt (**1**). The asymmetric unit of salt (**2**) shows a total of three C—H⋯I^−^ inter­molecular inter­actions with two neighboring mol­ecules (Fig. 2 ▸, Table 2 ▸). Two C—H moieties from the triazolium ring and one *ortho* C—H of the aryl ring inter­act with one iodide ion. In the asymmetric unit of salt (**3**) (Fig. 3 ▸, Table 3 ▸), there are a total of three C—H⋯I^−^ inter­molecular inter­actions, two from the triazolium C—H moieties, and one methine hydrogen atom from the isopropyl group because the aryl ring does not lie on the plane of the triazolium ring. For salts (**4**) and (**5**) (Figs. 4 ▸ and 5 ▸, Tables 4 ▸ and 5 ▸), there are only a total of two C—H⋯I/Br^−^ inter­molecular inter­actions, both from the triazole ring’s C—H groups. However, in salt (**5**), a water mol­ecule is in the asymmetric unit along the plane of the triazole and phenyl rings and is also inter­acting with the Br^−^ ion and the *ortho* C—H of the phenyl ring. A square-shaped hydrogen-bonding network is formed between two bromide ions and water mol­ecules (Fig. 6 ▸ and Table 5 ▸). Thus, each bromide ion has two acceptor inter­actions with water hydrogen atoms and one acceptor inter­action with the C—H of the triazolium ring, and each water mol­ecule has two donor inter­actions with the bromide ions and one acceptor inter­action with the *ortho* C—H of the aryl ring (Figs. 5 ▸ and 6 ▸, Table 5 ▸).

**Figure 7:**
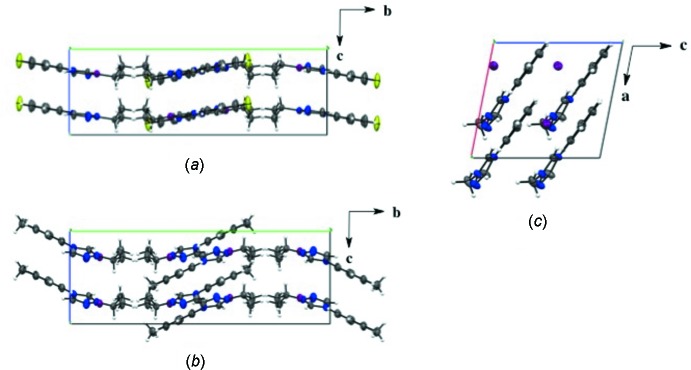
Layered structure observed in the packing of nearly flat cations with iodides. (*a*) Salt (**1**) viewed along the *a* axis; (*b*) salt (**2**) viewed along the *a* axis; and (*c*) salt (**4**) viewed along the *b* axis.

**Figure 8:**
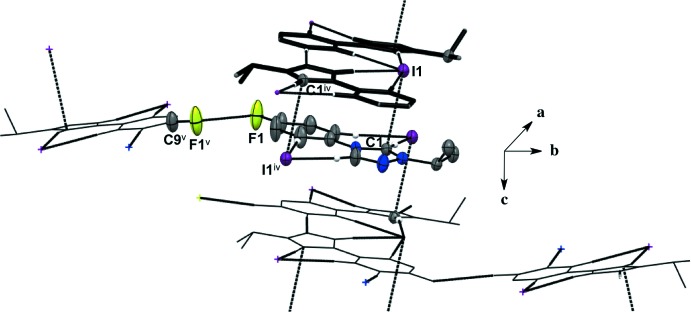
Salt (**1**) showing inter­molecular inter­actions between layers and neighboring cations. H atoms not participating in inter­molecular inter­actions are not shown. [Symmetry codes: (iv) −*x*, −*y*, −*z* + 1; (v) *x* − 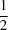, −*y* − 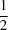, *z*.]

**Figure 9:**
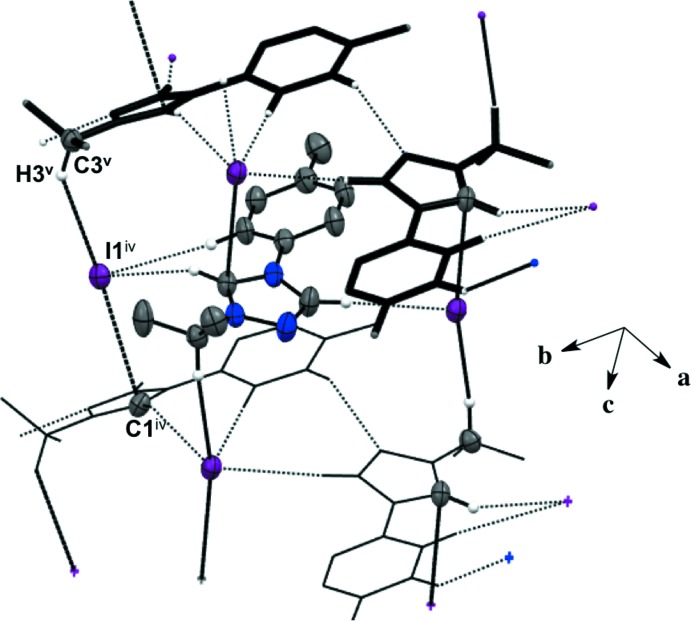
Salt (**2**) showing inter­molecular inter­actions between layers and neighboring cations. H atoms not participating in inter­molecular inter­actions are not shown. [Symmetry codes: (iv) −*x* + 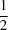, *y*, *z* + 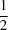; (v) −*x* + 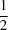, *y*, *z* − 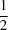.]

**Figure 10:**
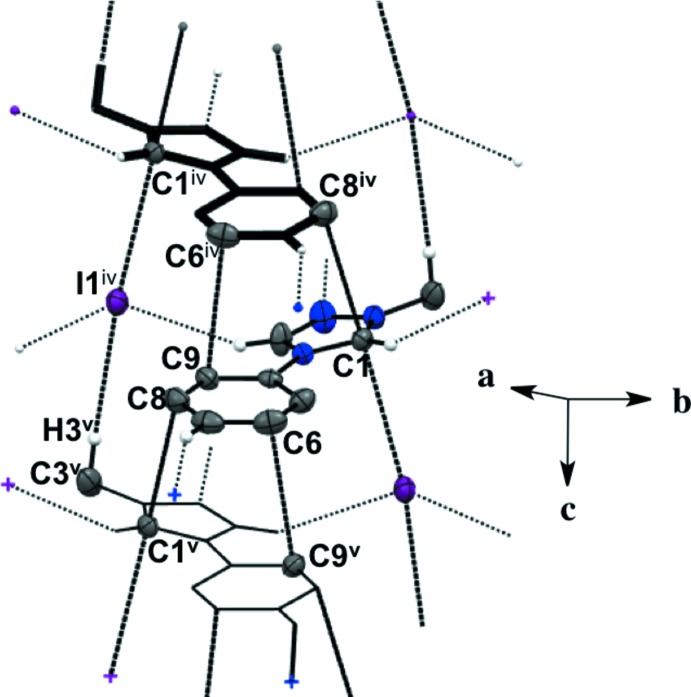
Salt (**4**) showing inter­molecular inter­actions between layers and neighboring cations as viewed along the *a* axis. H atoms not participating in inter­molecular inter­actions are not shown. [Symmetry codes: (iv) *x*, −*y*, *z* − 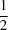; (v) *x*, −*y*, *z* + 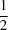.]

**Figure 12:**
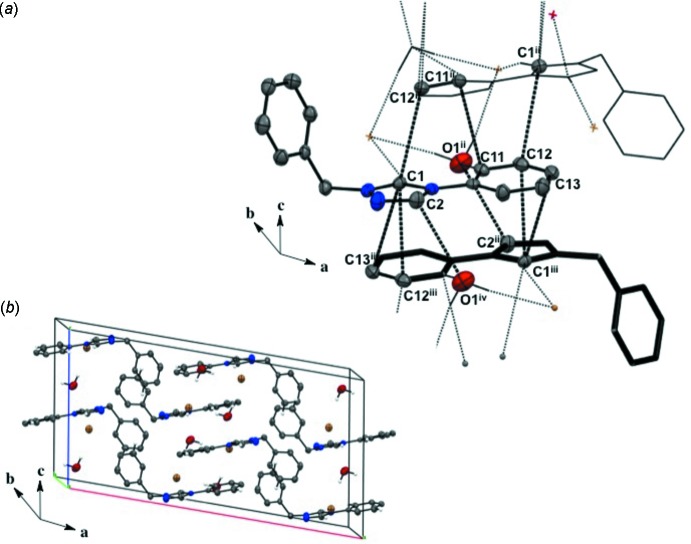
(*a*) Extended π–π inter­actions between triazolium and phenyl rings in salt (**5**). (*b*) Layered structure observed in the packing of nearly flat triazole and phenyl rings with a twisted benzyl ring of the cation in salt (**5**). H atoms not participating in the inter­actions are omitted. [Symmetry codes: (ii) −*x* + 2, *y*, −*z* + 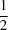; (iii) −*x* + 2, −*y*, −*z*; (iv) *x*, −*y*, *z* − 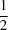.]

**Figure 13:**
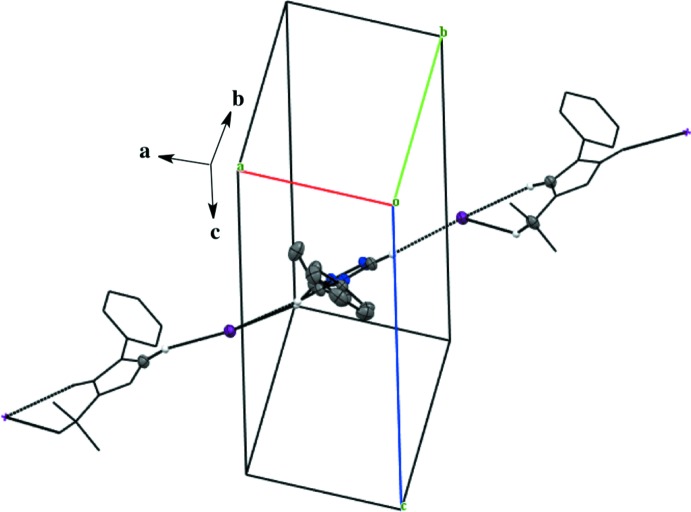
Extended sheet network in salt (**3**).

Salts (**1**), (**2**), and (**4**) pack as layered sheets as shown in Fig. 7 ▸. In salt (**1**), there is an additional inter­molecular inter­action between the triazolium carbon and the iodide ion (C1⋯I1) with a distance of 3.546 (4) Å between layers along the unit cell *c* axis (Fig. 8 ▸). While an anion–π inter­action is not common, similar inter­actions have been reported in the literature, especially in supra­molecular systems (Chifotides & Dunbar, 2013 ▸). Each cation in the sheet is further stabilized by an F⋯F inter­action with a distance of 2.889 (5) Å between neighboring cations (Fig. 8 ▸). C—F⋯F—C contacts are reported in the literature to be weak but still relevant for crystal packing (Chopra, 2012 ▸). In salt (**2**), the iodide ion between layers is inter­acting with both the triazolium carbon [C1⋯I1 distance of 3.532 (4) Å, Fig. 9 ▸] and the methine hydrogen atom of the isopropyl group (C3—H3⋯I1, Fig. 9 ▸, Table 2 ▸), in addition to the three hydrogen-bonding inter­actions with the *ortho* hydrogen atom and triazolium hydrogen atom of a cation within the sheet (Figs. 2 ▸ and 9 ▸, Table 2 ▸). Salt (**4**) also demonstrates iodide ion inter­action with both the triazolium carbon [C1⋯I1 distance of 3.503 (3) Å, Fig. 10 ▸] and the methyl hydrogen atom (C3—H3⋯I1; Fig. 10 ▸, Table 4 ▸) in alternating layers, in addition to the hydrogen bonding with the neighboring cation’s triazolium hydrogen atoms (Fig. 3 ▸, Table 4 ▸). The structure is stabilized further by π–π inter­actions between aryl carbon atoms in alternating layers [C6⋯C9 with a distance of 3.384 (5) Å] and an aryl carbon atom with a triazolium carbon atom [C1⋯C8 with a distance of 3.282 (4) Å], also in alternating layers (Fig. 10 ▸). In salt (**5**) there are π-inter­actions [C11⋯C11 with a distance of 3.220 (5) Å and C1⋯C12 with a distance of 3.335 (4) Å] between triazolium and aryl rings in alternating layers which are closely associated with the donor–acceptor inter­actions of the bromide ions and water mol­ecules (Figs. 6 ▸ and 11 ▸). Extending the layers further reveals another π–π inter­action [C1⋯C13 with a distance of 3.370 (4) Å] between the triazolium cation and aryl rings (Fig. 12 ▸), and a π–π inter­action [C2⋯O1, 3.143 (5) Å] between the carbon atom of the tri­azole ring and the oxygen atom of the water mol­ecule (Fig. 12 ▸ *a*). This triazole–phenyl π stacking is parallel with the *c* axis (Fig. 12 ▸ *b*). The extended sheet network in salt (**3**) passes diagonally through the cell, but there are no significant inter­molecular inter­actions between cations, as shown in Fig. 13 ▸.

Inter­estingly, when there is *para*-substitution on the aryl ring [salts (**1**) and (**2**)], there are no observed π–π inter­actions between the phenyl­ene and triazole rings. The observed inter­actions are predominantly from the triazolium carbon atom with the iodide ion. The absence of the *para*-substituents allows π–π inter­actions between the phenyl and triazole rings as demonstrated in salts (**4**) and (**5**). However, to facilitate π–π inter­action, the aryl ring needs to be co-planar with the triazolium ring; thus there are no π–π inter­actions in salt (**3**). Salt (**5**) exhibited the lowest melting-point temperature, possibly due to the presence of water in the crystal lattice, and thus will not be included in the discussion here. The higher melting points of salts (**1**), (**2**) and (**4**) compared to salt (**3**) may reflect the layering of the triazolium-aryl cation core sheets and the resulting inter-layer inter­actions. As predicted by Strassner, the electron-withdrawing substituent in the aryl ring found in salt (**1**) increased the melting point when compared to salt (**2**), which contains an electron-donating substituent on the aryl ring (Meyer & Strassner, 2011 ▸). The π–π inter­actions between the phenyl and triazole rings in salt (**4**) likely facilitate the increase in melting-point temperature.

In summary, for 1-alkyl-4-aryl-1,2,4-triazol-1-ium halide salts, the predominant inter­molecular inter­action is the C—H⋯halide hydrogen bond between the hydrogen atoms in the triazolium cation and the halide ions forming extended sheets. For salts with *para*-substitution on the aryl ring, π–π inter­actions between the triazolium carbon and the halide are present. The melting points of these salts agree with substit­uent inductive effects predictions. For salts without the *para*-substitution on the aryl ring, π–π inter­actions displayed by the layers are between the triazolium and aryl rings.

## Database survey   

Salt (**3**) is one of the azolium salts that was utilized by Abdellah in the direct electrochemical reduction of the salt to form the *N-*heterocyclic carbene (Abdellah *et al.*, 2011 ▸). Salt (**4**) is a carbene-precursor to phospho­rescent platinum(II)–NHC complexes; the crystal structure as a carbene ligand is also reported (Tenne *et al.*, 2013 ▸). Triazolium cation (**5**) was used in the investigation of kinetics and mechanism of azocoupling (Becker *et al.*, 1991 ▸).

## Synthesis and crystallization   

***General Methods***. All salts were synthesized in two steps. The first step is an intra­molecular transamination pathway similar to literature methods (Meyer & Strassner, 2011 ▸; Naik *et al.*, 2008 ▸; Holm *et al.*, 2010 ▸). The products of this transamination step are 4-(4-fluoro­phen­yl)-1,2,4-triazole as the salt (**1**) precursor, 4-(4-methyl­phen­yl)-1,2,4-triazole as the salt (**2**) precursor, and 4-(phen­yl)-1,2,4-triazole as salts (**3**), (**4**) and (**5**) precursor. In our attempts, we utilized a microwave reactor to shorten the reaction time from 24 hrs to roughly 15–30 mins with 20–70% yields (Meyer & Strassner, 2011 ▸; Naik *et al.*, 2008 ▸; Holm *et al.*, 2010 ▸). The second step is a nucleophilic substitution between the first-step products, 4-aryl-1,2,4-triazoles, and an alkyl halide (2-iodo­propane, iodo­methane, and benzyl bromide). This synthetic approach was used in the literature (Meyer & Strassner, 2011 ▸; Holm *et al.*, 2010 ▸), but in our attempts we again used the microwave reactor to shorten the reaction time from 48 hrs to 10-30 mins with 10-70% yields (Meyer & Strassner, 2011 ▸; Holm *et al.*, 2010 ▸).

*N*,*N*-di­methyl­formamide azine di­hydro­chloride (DMFA·2HCl) was synthesized following literature methods (Naik *et al.*, 2008 ▸; Holm *et al.*, 2010 ▸). All other reagents and solvents were purchased from Sigma-Aldrich. Tetra­hydro­furan (THF) and iso­propanol were dried with mol­ecular sieves (4Å). A Biotage microwave reactor was used for all synthetic preparations. All NMR spectra were recorded on a JEOL 400 MHz spectrometer. ^1^H and ^13^C NMR chemical shifts were determined by reference to residual ^1^H and ^13^C solvent peaks. All thermal analysis experiments were performed on a TA model TGA Q500 thermal gravimetric analyzer and TA model DSC Q100 differential scanning calorimeter. For TGA experiments, crystal samples with masses between 0.4 to 1.4 mg were loaded onto platinum pans. Dry grade nitro­gen gas was used for all samples with a balance purge rate of 40.00 mL/min and a sample purge rate of 60.00 mL/min. The temperature was ramped at 20.00 K per minute until a final temperature of 673.00 or 773.00 K was reached. For DSC experiments, crystal samples with masses between 3 and 9 mg were loaded onto platinum pans. Dry grade nitro­gen gas was used for all samples with a sample purge range of 50.00 mL/min. The samples were subjected to a heat/cool/heat cycle with a temperature ramp rate of 10.00 K per minute until a final temperature of 473–523 K was reached for the heating cycle, and a temperature ramp rate of 5.00 K per minute until a final temperature of 273 or 248 K was reached for the cooling cycle.

***Step 1: synthesis of 4-aryl-1,2,4-triazoles.*** A 20 mL microwave reaction vessel with a stir bar was charged with 1:1 molar equivalents of *N*,*N*-di­methyl­formamide azine di­hydro­chloride (DMFA·2HCl), and a *para-*substituted aryl amine (4-fluoro­aniline or *p*-toluidine), or aniline. The microwave was set to 443 or 453 K at normal absorbance, and run for 10–30 mins. Once completed, the mixture was washed with THF, dried with anhydrous magnesium sulfate and filtered. The solvent was removed *in vacuo*, and the remaining solid was washed with diethyl ether. ***Salt (1) precursor:*** **4-(4-fluoro­phen­yl)-1,2,4-triazole.** Brown oil (1.09 g, 72% yield).^1^H NMR (400 MHz, CDCl_3_): δ 8.44 (*s*, 2H, CH), 7.40–7.38 (*m*, 2H, Ar), 7.27–7.23 (*m*, 3H, Ar). ***Salt (2) precursor:*** **4-(4-methyl­phen­yl)-1,2,4-triazole.** Brown solid (0.26 g, 27% yield). ^1^H NMR (400 MHz, CDCl_3_): δ 8.45 (*s*, 2H, CH), 7.35–7.32 (*d*, 2H, Ar), 7.28–7.2 (*d*, 2H, Ar), 2.43 (*s*, 3H, Me). The proton spectrum values are the same as the literature values (Holm *et al.*, 2010 ▸). ***Salts (3), (4) and (5) precursor:*** **4-phenyl-1,2,4-triazole.** Brown solid (0.303 g, 22% yield). ^1^H NMR(400 MHz, CDCl_3_): δ 8.46 (*s*, 2H, CH), 7.54–7.49 (*m*, 2H, Ar) 7.47–7.42 (*m*, 1H, Ar), 7.39–7.36 (*m*, 2H, Ar). ^13^C NMR (101 MHz, CDCl_3_): δ 141.5, 133.9, 130.4, 129.1, 122.2. The proton and carbon spectra are the same as the literature values (Meyer & Strassner, 2011 ▸; Holm, *et al.*, 2010 ▸).

***Step 2: synthesis of 1-alkyl-4-aryl-1,2,4-triazole halides.*** A 20 mL microwave reaction vessel with a stir bar was charged with 1:2 molar equivalents of 4-aryl-1,2,4-triazole, a halide-substituted alkyl group (2-iodo­propane, iodo­methane, and benzyl bromide), and THF (5 mL). The microwave was set to 393–433 K at high absorbance for 10–30 mins. The resulting mixture was vacuum filtered, and washed with diethyl ether (3 × 10 mL). The solid product was recrystallized from hot iso­propanol and placed in the refrigerator for several days. **Salt (1): 1-isopropyl-4-(4-fluoro­phen­yl)-1,2,4-triazol-1-ium iodide.** Needle-like colorless crystals (0.070 g, 11% yield). ^1^H NMR (400 MHz, DMSO-*d*_6_): δ 10.70 (*s*, 1H, CH), 9.73 (*s*, 1H, CH), 7.94–7.90 (*dd*, 2H, Ar), 7.63–7.60 (*dd*, 2H, Ar), 4.84–4.82 (*sept*, 1H, *i*Pr), 1.61–1.59 (d, 6H, *i*Pr).^13^C NMR (101 MHz, DMSO-*d*_6_): δ 164.0, 161.5, 143.1, 140.8, 128.8, 125.5, 117.3, 117.1, 55.8, 21.3. Decomposition temp: 516.4 K. **Salt (2): 1-isopropyl-4-(4-methyl­phen­yl)-1,2,4-triazol-1-ium iodide.** Colorless prismatic crystals (0.22 g, 54% yield). ^1^H NMR (400 MHz, DMSO-*d*_6_): δ 10.68 (*s*, 1H, CH), 9.73 (*s*, 1H,) , 7.75–7.72 (*dd*, 2H, Ar), 7.71–7.50 (*d*, 2H, Ar), 4.88-4.78 (*sept*, 1H, *i*Pr), 2.41 (*s*, 3H, Me), 1.60–1.58 (d, 6H, *i*Pr).^13^C NMR (101 MHz, DMSO-*d*_6_): δ 142.7, 140.3, 140.2, 130.4, 129.8, 122.3, 55.6, 21.1, 20.7. Decomposition temp: 500.4 K. **Salt (3): 1-isopropyl-4-phenyl-1,2,4-triazol-1-ium iodide.** Colorless prismatic crystals (0.107 g, 24% yield). ^1^H NMR (400 MHz, DMSO-*d*_6_): δ 10.73 (*s*, 1H, CH), 9.77 (*s*, 1H, CH), 7.86–7.85 (*d*, 2H, Ar), 7.73–7.69 (*t*, 2H, Ar), 7.66–7.62 (*t*, 1H, Ar), 4.88–4.81 (*sept*, 1H, *i*Pr), 1.60–1.58 (*d*, 6H, Me). ^13^C NMR (101 MHz, DMSO-*d*_6_): δ 142.8, 140.4, 132.2, 130.5, 130.2, 122.6, 55.7, 21.2. Decomposition temp: 500.9 K. **Salt (4): 1-methyl-4-phenyl-1,2,4-triazol-1-ium iodide.** Colorless prism crystals (0.144 g, 70% yield). ^1^H NMR (400 MHz, DMSO-*d*_6_): δ 10.77 (*s*, 1H, CH), 9.76 (*s*, 1H, CH), 7.84–7.81 (*dt*, 2H, Ar), 7.73–7.66 (*tt*, 2H, Ar), 7.65–7.62 (*tt*, 1H, Ar), 4.15 (*s*, 3H, Me). ^13^C NMR (101 MHz, DMSO-*d*_6_): δ 142.7, 142.0, 132.1, 130.6, 130.3, 122.5, 39.0. Decomposition temp: 506.2 K.The proton and carbon spectroscopic values are the same as the literature values (Tenne *et al.*, 2013 ▸). **Salt (5): 1-benzyl-4-phenyl-1,2,4-triazol-1-ium bromide.** Colorless prismatic crystals (0.065 g, 10% yield).^1^H NMR (400 MHz, DMSO-*d*_6_) δ 11.05 (*s*, 1H, CH), 9.81 (*s*, 1H, CH), 7.87–7.84 (*dt*, 1H, Ar), 7.85–7.84 (*dd*, 1H, Ar), 7.72–7.68 (*tt*, 2H, Ar), 7.66–7.62 (*tt*, 1H, Ar), 7.56 (*m*, 2H, Bn), 7.47–7.41 (*m*, 3H, Bn), 5.71 (*s*, 2H, CH_2_). ^13^C NMR (101 MHz, DMSO-*d*_6_) δ 143.4, 141.9, 133.0, 132.2, 130.5, 130.2, 129.1, 129.0, 128.9, 122.6, 55.2. Decomposition temp: 431.8 K.

**Melting points:** salt (**1**), m.p.: 512.8 K; salt (**2**), m.p.: 489.4 K; salt (**3**), m.p.: 455.3 K; salt (**4**), m.p.: 505.7 K; salt (**5**), m.p.: 389.2 K.

## Refinement   

Crystal data, data collection and structure refinement details are summarized in Table 6 ▸. H atoms for salts (**1**)–(**4**) were placed in calculated positions and allowed to ride on their parent atoms at C—H distances of 0.95 Å for the triazolium and aryl rings, 0.98 Å for the methyl groups, and 1.00 Å for the methine group. H atoms for salt (**5**) were treated with a mixture of independent and constrained refinement. The C—H distances are 0.95 Å for the triazolium and aryl rings, 0.99 Å for the methyl­ene group, and 0.95 (6) Å and 0.92 (7) Å for water. Salt (**4**) crystallized in the non-centrosymmetric space group *Cc* with a Flack parameter of −0.01 (2) indicating the absolute structure is well determined.

## Supplementary Material

Crystal structure: contains datablock(s) salt1, salt2, salt3, salt4, salt5. DOI: 10.1107/S2056989015009019/zl2623sup1.cif

Structure factors: contains datablock(s) salt1. DOI: 10.1107/S2056989015009019/zl2623salt1sup2.hkl

Supporting information file. DOI: 10.1107/S2056989015009019/zl2623salt1sup7.cml

Structure factors: contains datablock(s) salt2. DOI: 10.1107/S2056989015009019/zl2623salt2sup3.hkl

Structure factors: contains datablock(s) salt3. DOI: 10.1107/S2056989015009019/zl2623salt3sup4.hkl

Structure factors: contains datablock(s) salt4. DOI: 10.1107/S2056989015009019/zl2623salt4sup5.hkl

Structure factors: contains datablock(s) salt5. DOI: 10.1107/S2056989015009019/zl2623salt5sup6.hkl

Supporting information file. DOI: 10.1107/S2056989015009019/zl2623salt2sup8.cml

Supporting information file. DOI: 10.1107/S2056989015009019/zl2623salt3sup9.cml

Supporting information file. DOI: 10.1107/S2056989015009019/zl2623salt4sup10.cml

Supporting information file. DOI: 10.1107/S2056989015009019/zl2623salt5sup11.cml

CCDC references: 1400159, 1400158, 1400157, 1400156, 1400155

Additional supporting information:  crystallographic information; 3D view; checkCIF report

## Figures and Tables

**Figure 11:**
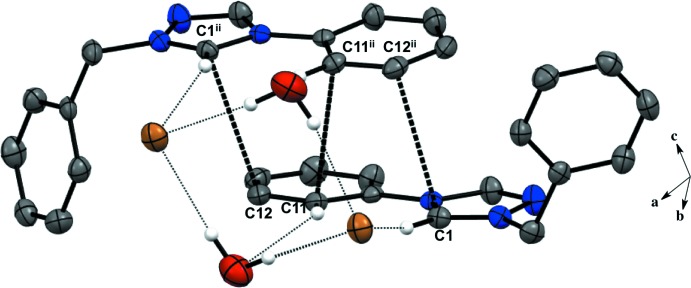
π–π inter­actions between the triazolium and phenyl rings in salt (**5**). H atoms not participating in the inter­actions are omitted. [Symmetry codes: (ii) −*x* + 2, *y*, −*z* + 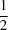; (iii) −*x* + 2, −*y*, −*z*; (iv) *x*, −*y*, *z* − 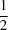.]

**Table 1:** Hydrogen-bond geometry (Å, °) for salt (**1**)

*D*—H⋯*A*	*D*—H	H⋯*A*	*D*⋯*A*	*D*—H⋯*A*
C1—H1⋯I1^i^	0.95	2.97	3.912 (4)	170
C2—H2⋯I1^ii^	0.95	2.83	3.774 (5)	173
C7—H7⋯I1^i^	0.95	2.86	3.801 (4)	170
C8—H8⋯N3^iii^	0.95	2.60	3.548 (6)	174
C11—H11⋯I1^ii^	0.95	3.13	4.083 (5)	177

Symmetry codes: (i) 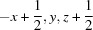; (ii) 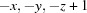; (iii) 
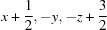
.

**Table 2:** Hydrogen-bond geometry (Å, °) for salt (**2**)

*D*—H⋯*A*	*D*—H	H⋯*A*	*D*⋯*A*	*D*—H⋯*A*
C1—H1⋯I1^i^	0.95	3.06	3.901 (3)	149
C2—H2⋯I1^ii^	0.95	2.84	3.771 (4)	168
C3—H3⋯I1^iii^	1.00	3.00	3.870 (4)	146
C11—H11⋯I1^i^	0.95	2.98	3.930 (3)	174

Symmetry codes: (i) 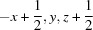; (ii) 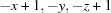; (iii) 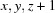.

**Table 3:** Hydrogen-bond geometry (Å, °) for salt (**3**)

*D*—H⋯*A*	*D*—H	H⋯*A*	*D*⋯*A*	*D*—H⋯*A*
C1—H1⋯I1^i^	0.95	2.87	3.744 (2)	153
C2—H2⋯I1^ii^	0.95	2.94	3.800 (2)	151
C3—H3⋯I1^i^	1.00	3.18	4.033 (2)	145

Symmetry codes: (i) 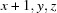; (ii) 
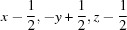
.

**Table 4:** Hydrogen-bond geometry (Å, °) for salt (**4**)

*D*—H⋯*A*	*D*—H	H⋯*A*	*D*⋯*A*	*D*—H⋯*A*
C1—H1⋯I1^i^	0.95	2.85	3.707 (4)	150
C2—H2⋯I1^ii^	0.95	2.94	3.811 (4)	153
C3—H3*B*⋯I1^iii^	0.98	3.10	4.079 (6)	176

Symmetry codes: (i) 
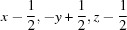
; (ii) 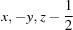; (iii) 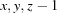.

**Table 5:** Hydrogen-bond geometry (Å, °) for salt (**5**)

*D*—H⋯*A*	*D*—H	H⋯*A*	*D*⋯*A*	*D*—H⋯*A*
C1—H1⋯Br1	0.95	2.59	3.455 (3)	151
C2—H2⋯Br1^i^	0.95	2.75	3.644 (3)	156
C11—H11⋯O1^ii^	0.95	2.55	3.247 (4)	130
O1—H1*A*⋯Br1^ii^	0.95 (6)	2.42 (6)	3.365 (3)	172 (4)
O1—H1*B*⋯Br1	0.92 (7)	2.43 (7)	3.341 (3)	170 (5)

Symmetry codes: (i) 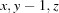; (ii) 
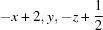
.

**Table:** 

	Salt (**1**)	Salt (**2**)	Salt (**3**)
Crystal data			
Chemical formula	C_11_H_13_FN_3_^+^·I^−^	C_12_H_16_N_3_^+^·I^−^	C_11_H_14_N_3_^+^·I^−^
*M*_r_	333.14	329.18	315.15
Crystal system, space group	Orthorhombic, *P* *c* *c* *n*	Orthorhombic, *P* *c* *c* *n*	Monoclinic, *P*2_1_/*n*
Temperature (K)	173	173	173
*a*, *b*, *c* (Å)	16.396 (3), 21.732 (4), 7.2412 (12)	15.843 (3), 21.933 (4), 7.8250 (14)	5.9326 (11), 17.826 (3), 12.129 (2)
α, β, γ (°)	90, 90, 90	90, 90, 90	90, 102.897 (7), 90
*V* (Å^3^)	2580.1 (7)	2719.0 (8)	1250.3 (4)
*Z*	8	8	4
Radiation type	Mo *K*α	Mo *K*α	Mo *K*α
μ (mm^−1^)	2.47	2.34	2.54
Crystal size (mm)	0.71 × 0.05 × 0.02	0.52 × 0.12 × 0.04	0.80 × 0.40 × 0.10

Data collection			
Diffractometer	Rigaku XtaLAB mini	Rigaku XtaLAB mini	Rigaku XtaLAB mini
Absorption correction	Multi-scan (*REQAB*; Rigaku, 1998 ▸)	Multi-scan (*REQAB*; Rigaku, 1998 ▸)	Multi-scan (*REQAB*; Rigaku, 1998 ▸)
*T*_min_, *T*_max_	0.671, 0.952	0.564, 0.911	0.356, 0.776
No. of measured, independent and observed [*I* > 2σ(*I*)] reflections	17829, 2632, 1872	16099, 2767, 2150	12823, 2858, 2582
*R*_int_	0.073	0.051	0.044
(sin θ/λ)_max_ (Å^−1^)	0.625	0.625	0.649

Refinement			
*R*[*F* ^2^ > 2σ(*F* ^2^)], *wR*(*F* ^2^), *S*	0.035, 0.077, 1.03	0.031, 0.065, 1.04	0.023, 0.055, 1.09
No. of reflections	2632	2767	2858
No. of parameters	147	148	138
No. of restraints	0	0	0
H-atom treatment	H-atom parameters constrained	H-atom parameters constrained	H-atom parameters constrained
Δρ_max_, Δρ_min_ (e Å^−3^)	0.60, −0.58	0.43, −0.36	0.40, −0.69

**Table:** 

	Salt (**4**)	Salt (**5**)
Crystal data		
Chemical formula	C_9_H_10_N_3_^+^·I^−^	C_15_H_14_N_3_^+^·Br^−^·H_2_O
*M*_r_	287.10	334.22
Crystal system, space group	Monoclinic, *C* *c*	Monoclinic, *C*2/*c*
Temperature (K)	173	173
*a*, *b*, *c* (Å)	7.660 (2), 16.912 (5), 8.412 (3)	24.783 (6), 8.996 (2), 13.089 (3)
α, β, γ (°)	90, 101.137 (7), 90	90, 100.068 (7), 90
*V* (Å^3^)	1069.2 (6)	2873.3 (13)
*Z*	4	8
Radiation type	Mo *K*α	Mo *K*α
μ (mm^−1^)	2.96	2.86
Crystal size (mm)	0.31 × 0.23 × 0.13	0.60 × 0.37 × 0.17

Data collection		
Diffractometer	Rigaku XtaLAB mini	Rigaku XtaLAB mini
Absorption correction	Multi-scan (*REQAB*; Rigaku, 1998 ▸)	Multi-scan (*REQAB*; Rigaku, 1998 ▸)
*T*_min_, *T*_max_	0.439, 0.681	0.321, 0.614
No. of measured, independent and observed [*I* > 2σ(*I*)] reflections	5472, 2420, 2359	6672, 3259, 2630
*R*_int_	0.020	0.031
(sin θ/λ)_max_ (Å^−1^)	0.649	0.650

Refinement		
*R*[*F* ^2^ > 2σ(*F* ^2^)], *wR*(*F* ^2^), *S*	0.018, 0.037, 1.05	0.043, 0.098, 1.08
No. of reflections	2420	3259
No. of parameters	119	189
No. of restraints	2	0
H-atom treatment	H-atom parameters constrained	H atoms treated by a mixture of independent and constrained refinement
Δρ_max_, Δρ_min_ (e Å^−3^)	0.18, −0.36	0.75, −0.63
Absolute structure	Flack *x* determined using 1112 quotients [(*I* ^+^)−(*I* ^−^)]/[(*I* ^+^)+(*I* ^−^)] (Parsons et al., 2013 ▸)	–
Absolute structure parameter	−0.012 (18)	–

Computer programs: *CrystalClearSM Expert* (Rigaku, 2011 ▸), *SIR97* (Altomare *et al.*, 1999 ▸), *SHELXL2014* (Sheldrick, 2015 ▸), *CrystalStructure* (Rigaku, 2010 ▸) and *Mercury* (Macrae *et al.*, 2006 ▸).

## supplementary crystallographic information

### (salt1) 4-(4-Fluorophenyl)-1-isopropyl-1,2,4-triazol-1-ium iodide Crystal data

**Table:**

C_11_H_13_FN_3_^+^·I^−^	*D*_x_ = 1.715 Mg m^−^^3^
*M**_r_* = 333.14	Mo *K*α radiation, λ = 0.71075 Å
Orthorhombic, *P**c**c**n*	Cell parameters from 13432 reflections
*a* = 16.396 (3) Å	θ = 3.1–26.5°
*b* = 21.732 (4) Å	µ = 2.47 mm^−^^1^
*c* = 7.2412 (12) Å	*T* = 173 K
*V* = 2580.1 (7) Å^3^	Needle, colorless
*Z* = 8	0.71 × 0.05 × 0.02 mm
*F*(000) = 1296	

### (salt1) 4-(4-Fluorophenyl)-1-isopropyl-1,2,4-triazol-1-ium iodide Data collection

**Table:**

Rigaku XtaLAB mini diffractometer	1872 reflections with *I* > 2σ(*I*)
Detector resolution: 6.849 pixels mm^-1^	*R*_int_ = 0.073
ω scans	θ_max_ = 26.4°, θ_min_ = 3.1°
Absorption correction: multi-scan (*REQAB*; Rigaku, 1998)	*h* = −16→20
*T*_min_ = 0.671, *T*_max_ = 0.952	*k* = −27→27
17829 measured reflections	*l* = −9→9
2632 independent reflections	

### (salt1) 4-(4-Fluorophenyl)-1-isopropyl-1,2,4-triazol-1-ium iodide Refinement

**Table:**

Refinement on *F*^2^	0 restraints
Least-squares matrix: full	Hydrogen site location: inferred from neighbouring sites
*R*[*F*^2^ > 2σ(*F*^2^)] = 0.035	H-atom parameters constrained
*wR*(*F*^2^) = 0.077	*w* = 1/[σ^2^(*F*_o_^2^) + (0.0311*P*)^2^ + 1.7321*P*] where *P* = (*F*_o_^2^ + 2*F*_c_^2^)/3
*S* = 1.03	(Δ/σ)_max_ < 0.001
2632 reflections	Δρ_max_ = 0.60 e Å^−^^3^
147 parameters	Δρ_min_ = −0.58 e Å^−^^3^

### (salt1) 4-(4-Fluorophenyl)-1-isopropyl-1,2,4-triazol-1-ium iodide Special details

**Table:**

Geometry. All e.s.d.'s (except the e.s.d. in the dihedral angle between two l.s. planes) are estimated using the full covariance matrix. The cell e.s.d.'s are taken into account individually in the estimation of e.s.d.'s in distances, angles and torsion angles; correlations between e.s.d.'s in cell parameters are only used when they are defined by crystal symmetry. An approximate (isotropic) treatment of cell e.s.d.'s is used for estimating e.s.d.'s involving l.s. planes.

### (salt1) 4-(4-Fluorophenyl)-1-isopropyl-1,2,4-triazol-1-ium iodide Fractional atomic coordinates and isotropic or equivalent isotropic displacement parameters (Å^2^)

**Table:**

	*x*	*y*	*z*	*U*_iso_*/*U*_eq_	
I1	0.12779 (2)	0.10735 (2)	0.28479 (4)	0.03130 (11)	
F1	0.2967 (2)	−0.19361 (13)	0.6333 (6)	0.0857 (12)	
N1	0.1110 (2)	0.01458 (15)	0.7471 (4)	0.0293 (8)	
N2	0.0756 (2)	0.10799 (15)	0.7882 (5)	0.0304 (8)	
N3	0.0050 (2)	0.07473 (17)	0.7909 (6)	0.0419 (10)	
C1	0.1383 (3)	0.07255 (19)	0.7626 (5)	0.0287 (10)	
H1	0.1936	0.0854	0.7559	0.034*	
C2	0.0290 (3)	0.0188 (2)	0.7647 (7)	0.0427 (12)	
H2	−0.0069	−0.0155	0.7584	0.051*	
C3	0.0723 (3)	0.17592 (19)	0.8200 (6)	0.0313 (10)	
H3	0.0550	0.1833	0.9506	0.038*	
C4	0.0089 (3)	0.2039 (2)	0.6936 (7)	0.0426 (12)	
H4A	−0.0445	0.1859	0.7206	0.051*	
H4B	0.0235	0.1955	0.5649	0.051*	
H4C	0.0068	0.2485	0.7136	0.051*	
C5	0.1571 (3)	0.2027 (2)	0.7944 (7)	0.0398 (11)	
H5A	0.1764	0.1937	0.6692	0.048*	
H5B	0.1944	0.1843	0.8846	0.048*	
H5C	0.1552	0.2473	0.8129	0.048*	
C6	0.1595 (3)	−0.0401 (2)	0.7140 (6)	0.0338 (10)	
C7	0.2430 (3)	−0.03589 (19)	0.7138 (7)	0.0405 (12)	
H7	0.2687	0.0027	0.7333	0.049*	
C8	0.2895 (3)	−0.0880 (2)	0.6851 (7)	0.0472 (13)	
H8	0.3474	−0.0857	0.6827	0.057*	
C9	0.2503 (3)	−0.1427 (2)	0.6601 (8)	0.0533 (14)	
C10	0.1678 (3)	−0.1476 (2)	0.6597 (9)	0.0651 (17)	
H10	0.1426	−0.1864	0.6401	0.078*	
C11	0.1205 (3)	−0.0954 (2)	0.6883 (8)	0.0515 (14)	
H11	0.0626	−0.0979	0.6899	0.062*	

### (salt1) 4-(4-Fluorophenyl)-1-isopropyl-1,2,4-triazol-1-ium iodide Atomic displacement parameters (Å^2^)

**Table:**

	*U*^11^	*U*^22^	*U*^33^	*U*^12^	*U*^13^	*U*^23^
I1	0.03225 (18)	0.02454 (15)	0.03710 (17)	−0.00037 (12)	0.00019 (13)	−0.00025 (12)
F1	0.054 (2)	0.0306 (16)	0.173 (4)	0.0116 (15)	−0.011 (2)	−0.010 (2)
N1	0.031 (2)	0.0197 (16)	0.037 (2)	−0.0012 (15)	−0.0021 (15)	0.0028 (14)
N2	0.037 (2)	0.0243 (18)	0.0300 (18)	−0.0021 (16)	−0.0007 (16)	−0.0009 (17)
N3	0.027 (2)	0.030 (2)	0.069 (3)	−0.0055 (16)	0.0044 (19)	−0.007 (2)
C1	0.031 (3)	0.024 (2)	0.031 (2)	−0.0044 (18)	0.0006 (17)	−0.0012 (17)
C2	0.036 (3)	0.025 (2)	0.067 (4)	−0.006 (2)	0.000 (2)	−0.003 (2)
C3	0.039 (3)	0.023 (2)	0.032 (3)	0.0029 (19)	0.0006 (18)	−0.0027 (19)
C4	0.045 (3)	0.032 (2)	0.051 (3)	0.008 (2)	−0.001 (2)	0.001 (2)
C5	0.038 (3)	0.027 (2)	0.055 (3)	−0.0022 (19)	−0.003 (2)	−0.004 (2)
C6	0.031 (3)	0.027 (2)	0.043 (3)	0.0011 (19)	−0.003 (2)	0.000 (2)
C7	0.037 (3)	0.022 (2)	0.062 (3)	−0.005 (2)	0.000 (2)	−0.001 (2)
C8	0.033 (3)	0.035 (3)	0.073 (4)	−0.002 (2)	−0.001 (2)	−0.001 (2)
C9	0.044 (3)	0.028 (3)	0.089 (4)	0.004 (2)	−0.011 (3)	−0.004 (3)
C10	0.042 (4)	0.024 (3)	0.129 (5)	−0.004 (2)	−0.010 (3)	−0.003 (3)
C11	0.027 (3)	0.033 (3)	0.094 (4)	−0.004 (2)	−0.010 (3)	−0.001 (3)

### (salt1) 4-(4-Fluorophenyl)-1-isopropyl-1,2,4-triazol-1-ium iodide Geometric parameters (Å, º)

**Table:**

F1—C9	1.356 (6)	C4—H4C	0.9800
N1—C1	1.342 (5)	C5—H5A	0.9800
N1—C2	1.354 (6)	C5—H5B	0.9800
N1—C6	1.450 (6)	C5—H5C	0.9800
N2—C1	1.298 (5)	C6—C7	1.371 (6)
N2—N3	1.365 (5)	C6—C11	1.375 (6)
N2—C3	1.495 (5)	C7—C8	1.381 (6)
N3—C2	1.292 (6)	C7—H7	0.9500
C1—H1	0.9500	C8—C9	1.364 (7)
C2—H2	0.9500	C8—H8	0.9500
C3—C4	1.512 (6)	C9—C10	1.358 (7)
C3—C5	1.519 (6)	C10—C11	1.390 (7)
C3—H3	1.0000	C10—H10	0.9500
C4—H4A	0.9800	C11—H11	0.9500
C4—H4B	0.9800		
			
C1—N1—C2	105.1 (4)	C3—C5—H5A	109.5
C1—N1—C6	126.9 (4)	C3—C5—H5B	109.5
C2—N1—C6	128.0 (4)	H5A—C5—H5B	109.5
C1—N2—N3	111.1 (3)	C3—C5—H5C	109.5
C1—N2—C3	129.5 (4)	H5A—C5—H5C	109.5
N3—N2—C3	119.3 (3)	H5B—C5—H5C	109.5
C2—N3—N2	103.8 (4)	C7—C6—C11	121.6 (4)
N2—C1—N1	107.7 (4)	C7—C6—N1	119.5 (4)
N2—C1—H1	126.1	C11—C6—N1	118.9 (4)
N1—C1—H1	126.1	C6—C7—C8	119.8 (4)
N3—C2—N1	112.3 (4)	C6—C7—H7	120.1
N3—C2—H2	123.9	C8—C7—H7	120.1
N1—C2—H2	123.9	C9—C8—C7	118.3 (5)
N2—C3—C4	109.2 (3)	C9—C8—H8	120.9
N2—C3—C5	109.0 (3)	C7—C8—H8	120.9
C4—C3—C5	113.6 (4)	F1—C9—C10	119.6 (5)
N2—C3—H3	108.3	F1—C9—C8	117.8 (5)
C4—C3—H3	108.3	C10—C9—C8	122.6 (5)
C5—C3—H3	108.3	C9—C10—C11	119.5 (5)
C3—C4—H4A	109.5	C9—C10—H10	120.3
C3—C4—H4B	109.5	C11—C10—H10	120.3
H4A—C4—H4B	109.5	C6—C11—C10	118.3 (5)
C3—C4—H4C	109.5	C6—C11—H11	120.9
H4A—C4—H4C	109.5	C10—C11—H11	120.9
H4B—C4—H4C	109.5		
			
C1—N2—N3—C2	−0.4 (5)	C2—N1—C6—C7	−175.2 (5)
C3—N2—N3—C2	−178.2 (4)	C1—N1—C6—C11	−175.1 (4)
N3—N2—C1—N1	0.1 (5)	C2—N1—C6—C11	3.1 (7)
C3—N2—C1—N1	177.7 (4)	C11—C6—C7—C8	0.7 (8)
C2—N1—C1—N2	0.2 (4)	N1—C6—C7—C8	179.0 (4)
C6—N1—C1—N2	178.8 (4)	C6—C7—C8—C9	−0.9 (8)
N2—N3—C2—N1	0.6 (5)	C7—C8—C9—F1	−179.6 (5)
C1—N1—C2—N3	−0.5 (5)	C7—C8—C9—C10	1.0 (9)
C6—N1—C2—N3	−179.0 (4)	F1—C9—C10—C11	179.6 (6)
C1—N2—C3—C4	132.0 (5)	C8—C9—C10—C11	−1.0 (10)
N3—N2—C3—C4	−50.6 (5)	C7—C6—C11—C10	−0.6 (8)
C1—N2—C3—C5	7.3 (6)	N1—C6—C11—C10	−178.9 (5)
N3—N2—C3—C5	−175.3 (4)	C9—C10—C11—C6	0.7 (9)
C1—N1—C6—C7	6.5 (7)		

### (salt1) 4-(4-Fluorophenyl)-1-isopropyl-1,2,4-triazol-1-ium iodide Hydrogen-bond geometry (Å, º)

**Table:**

*D*—H···*A*	*D*—H	H···*A*	*D*···*A*	*D*—H···*A*
C1—H1···I1^i^	0.95	2.97	3.912 (4)	170
C2—H2···I1^ii^	0.95	2.83	3.774 (5)	173
C7—H7···I1^i^	0.95	2.86	3.801 (4)	170
C8—H8···N3^iii^	0.95	2.60	3.548 (6)	174
C11—H11···I1^ii^	0.95	3.13	4.083 (5)	177

Symmetry codes: (i) −*x*+1/2, *y*, *z*+1/2; (ii) −*x*, −*y*, −*z*+1; (iii) *x*+1/2, −*y*, −*z*+3/2.

### (salt2) 1-Isopropyl-4-(4-methylphenyl)-1,2,4-triazol-1-ium iodide Crystal data

**Table:**

C_12_H_16_N_3_^+^·I^−^	*D*_x_ = 1.608 Mg m^−^^3^
*M**_r_* = 329.18	Mo *K*α radiation, λ = 0.71075 Å
Orthorhombic, *P**c**c**n*	Cell parameters from 12738 reflections
*a* = 15.843 (3) Å	θ = 3.1–26.4°
*b* = 21.933 (4) Å	µ = 2.34 mm^−^^1^
*c* = 7.8250 (14) Å	*T* = 173 K
*V* = 2719.0 (8) Å^3^	Prism, colorless
*Z* = 8	0.52 × 0.12 × 0.04 mm
*F*(000) = 1296	

### (salt2) 1-Isopropyl-4-(4-methylphenyl)-1,2,4-triazol-1-ium iodide Data collection

**Table:**

Rigaku XtaLAB mini diffractometer	2150 reflections with *I* > 2σ(*I*)
Detector resolution: 6.849 pixels mm^-1^	*R*_int_ = 0.051
ω scans	θ_max_ = 26.4°, θ_min_ = 3.1°
Absorption correction: multi-scan (*REQAB*; Rigaku, 1998)	*h* = −17→19
*T*_min_ = 0.564, *T*_max_ = 0.911	*k* = −27→27
16099 measured reflections	*l* = −9→9
2767 independent reflections	

### (salt2) 1-Isopropyl-4-(4-methylphenyl)-1,2,4-triazol-1-ium iodide Refinement

**Table:**

Refinement on *F*^2^	0 restraints
Least-squares matrix: full	Hydrogen site location: inferred from neighbouring sites
*R*[*F*^2^ > 2σ(*F*^2^)] = 0.031	H-atom parameters constrained
*wR*(*F*^2^) = 0.065	*w* = 1/[σ^2^(*F*_o_^2^) + (0.0241*P*)^2^ + 1.7237*P*] where *P* = (*F*_o_^2^ + 2*F*_c_^2^)/3
*S* = 1.04	(Δ/σ)_max_ = 0.001
2767 reflections	Δρ_max_ = 0.43 e Å^−^^3^
148 parameters	Δρ_min_ = −0.36 e Å^−^^3^

### (salt2) 1-Isopropyl-4-(4-methylphenyl)-1,2,4-triazol-1-ium iodide Special details

**Table:**

Geometry. All e.s.d.'s (except the e.s.d. in the dihedral angle between two l.s. planes) are estimated using the full covariance matrix. The cell e.s.d.'s are taken into account individually in the estimation of e.s.d.'s in distances, angles and torsion angles; correlations between e.s.d.'s in cell parameters are only used when they are defined by crystal symmetry. An approximate (isotropic) treatment of cell e.s.d.'s is used for estimating e.s.d.'s involving l.s. planes.

### (salt2) 1-Isopropyl-4-(4-methylphenyl)-1,2,4-triazol-1-ium iodide Fractional atomic coordinates and isotropic or equivalent isotropic displacement parameters (Å^2^)

**Table:**

	*x*	*y*	*z*	*U*_iso_*/*U*_eq_	
I1	0.39750 (2)	0.11566 (2)	0.28064 (3)	0.03506 (9)	
N1	0.36095 (16)	0.01570 (12)	0.6774 (4)	0.0323 (7)	
N2	0.40416 (16)	0.10301 (12)	0.7635 (4)	0.0323 (7)	
N3	0.47092 (18)	0.06448 (14)	0.7832 (4)	0.0474 (8)	
C1	0.3387 (2)	0.07437 (14)	0.7003 (4)	0.0327 (8)	
H1	0.2852	0.0918	0.6752	0.039*	
C2	0.4431 (2)	0.01195 (17)	0.7293 (5)	0.0466 (10)	
H2	0.4758	−0.0243	0.7266	0.056*	
C3	0.4142 (2)	0.16834 (15)	0.8098 (5)	0.0360 (8)	
H3	0.4272	0.1708	0.9347	0.043*	
C4	0.3329 (2)	0.20152 (16)	0.7779 (6)	0.0558 (12)	
H4A	0.2877	0.1826	0.8450	0.067*	
H4B	0.3188	0.1993	0.6561	0.067*	
H4C	0.3391	0.2443	0.8117	0.067*	
C5	0.4878 (2)	0.19528 (16)	0.7123 (5)	0.0483 (10)	
H5A	0.4763	0.1933	0.5894	0.058*	
H5B	0.5392	0.1721	0.7379	0.058*	
H5C	0.4958	0.2379	0.7465	0.058*	
C6	0.3098 (2)	−0.03293 (14)	0.6067 (4)	0.0335 (8)	
C7	0.3493 (2)	−0.08364 (16)	0.5362 (5)	0.0417 (9)	
H7	0.4090	−0.0858	0.5296	0.050*	
C8	0.3000 (2)	−0.13047 (15)	0.4763 (5)	0.0449 (10)	
H8	0.3269	−0.1655	0.4301	0.054*	
C9	0.2121 (2)	−0.12853 (15)	0.4805 (5)	0.0387 (9)	
C10	0.1751 (2)	−0.07632 (15)	0.5485 (5)	0.0401 (9)	
H10	0.1153	−0.0731	0.5506	0.048*	
C11	0.2231 (2)	−0.02902 (14)	0.6130 (5)	0.0377 (9)	
H11	0.1966	0.0058	0.6612	0.045*	
C12	0.1601 (3)	−0.18091 (17)	0.4159 (6)	0.0535 (11)	
H12A	0.1066	−0.1655	0.3703	0.064*	
H12B	0.1487	−0.2093	0.5099	0.064*	
H12C	0.1910	−0.2022	0.3252	0.064*	

### (salt2) 1-Isopropyl-4-(4-methylphenyl)-1,2,4-triazol-1-ium iodide Atomic displacement parameters (Å^2^)

**Table:**

	*U*^11^	*U*^22^	*U*^33^	*U*^12^	*U*^13^	*U*^23^
I1	0.03260 (14)	0.02809 (12)	0.04449 (16)	0.00250 (9)	−0.00283 (10)	0.00031 (10)
N1	0.0298 (15)	0.0265 (14)	0.0406 (18)	0.0021 (11)	0.0055 (13)	0.0034 (12)
N2	0.0265 (15)	0.0293 (14)	0.0411 (18)	0.0028 (11)	0.0026 (13)	−0.0011 (12)
N3	0.0322 (16)	0.0385 (17)	0.071 (2)	0.0074 (13)	−0.0089 (17)	−0.0046 (16)
C1	0.0283 (17)	0.0266 (16)	0.043 (2)	0.0031 (14)	0.0050 (16)	0.0047 (15)
C2	0.036 (2)	0.036 (2)	0.068 (3)	0.0101 (17)	−0.003 (2)	−0.0004 (19)
C3	0.038 (2)	0.0299 (18)	0.040 (2)	−0.0017 (14)	0.0005 (16)	−0.0045 (15)
C4	0.039 (2)	0.0287 (19)	0.100 (4)	0.0033 (16)	0.004 (2)	−0.005 (2)
C5	0.039 (2)	0.042 (2)	0.064 (3)	−0.0091 (17)	0.006 (2)	−0.006 (2)
C6	0.0343 (19)	0.0263 (17)	0.040 (2)	0.0014 (15)	0.0069 (17)	0.0046 (14)
C7	0.034 (2)	0.035 (2)	0.057 (3)	0.0052 (16)	0.0123 (18)	−0.0020 (17)
C8	0.047 (2)	0.028 (2)	0.060 (3)	0.0077 (16)	0.015 (2)	−0.0016 (16)
C9	0.042 (2)	0.0297 (19)	0.044 (2)	−0.0033 (15)	0.0061 (18)	0.0026 (15)
C10	0.0319 (19)	0.037 (2)	0.051 (2)	0.0029 (16)	0.0060 (18)	0.0005 (17)
C11	0.035 (2)	0.0264 (17)	0.051 (3)	0.0067 (14)	0.0062 (18)	−0.0030 (16)
C12	0.055 (2)	0.041 (2)	0.065 (3)	−0.0058 (19)	0.012 (2)	−0.006 (2)

### (salt2) 1-Isopropyl-4-(4-methylphenyl)-1,2,4-triazol-1-ium iodide Geometric parameters (Å, º)

**Table:**

N1—C1	1.346 (4)	C5—H5B	0.9800
N1—C2	1.365 (4)	C5—H5C	0.9800
N1—C6	1.449 (4)	C6—C11	1.377 (4)
N2—C1	1.310 (4)	C6—C7	1.390 (4)
N2—N3	1.363 (4)	C7—C8	1.372 (5)
N2—C3	1.487 (4)	C7—H7	0.9500
N3—C2	1.304 (5)	C8—C9	1.394 (5)
C1—H1	0.9500	C8—H8	0.9500
C2—H2	0.9500	C9—C10	1.392 (5)
C3—C4	1.500 (5)	C9—C12	1.502 (5)
C3—C5	1.514 (5)	C10—C11	1.383 (5)
C3—H3	1.0000	C10—H10	0.9500
C4—H4A	0.9800	C11—H11	0.9500
C4—H4B	0.9800	C12—H12A	0.9800
C4—H4C	0.9800	C12—H12B	0.9800
C5—H5A	0.9800	C12—H12C	0.9800
			
C1—N1—C2	105.5 (3)	C3—C5—H5C	109.5
C1—N1—C6	127.5 (3)	H5A—C5—H5C	109.5
C2—N1—C6	127.0 (3)	H5B—C5—H5C	109.5
C1—N2—N3	111.1 (3)	C11—C6—C7	120.8 (3)
C1—N2—C3	129.7 (3)	C11—C6—N1	119.9 (3)
N3—N2—C3	119.1 (3)	C7—C6—N1	119.3 (3)
C2—N3—N2	104.4 (3)	C8—C7—C6	118.6 (3)
N2—C1—N1	107.5 (3)	C8—C7—H7	120.7
N2—C1—H1	126.2	C6—C7—H7	120.7
N1—C1—H1	126.2	C7—C8—C9	122.5 (3)
N3—C2—N1	111.4 (3)	C7—C8—H8	118.8
N3—C2—H2	124.3	C9—C8—H8	118.8
N1—C2—H2	124.3	C10—C9—C8	117.1 (3)
N2—C3—C4	109.6 (3)	C10—C9—C12	121.8 (3)
N2—C3—C5	109.6 (3)	C8—C9—C12	121.1 (3)
C4—C3—C5	112.8 (3)	C11—C10—C9	121.6 (3)
N2—C3—H3	108.2	C11—C10—H10	119.2
C4—C3—H3	108.2	C9—C10—H10	119.2
C5—C3—H3	108.2	C6—C11—C10	119.3 (3)
C3—C4—H4A	109.5	C6—C11—H11	120.3
C3—C4—H4B	109.5	C10—C11—H11	120.3
H4A—C4—H4B	109.5	C9—C12—H12A	109.5
C3—C4—H4C	109.5	C9—C12—H12B	109.5
H4A—C4—H4C	109.5	H12A—C12—H12B	109.5
H4B—C4—H4C	109.5	C9—C12—H12C	109.5
C3—C5—H5A	109.5	H12A—C12—H12C	109.5
C3—C5—H5B	109.5	H12B—C12—H12C	109.5
H5A—C5—H5B	109.5		
			
C1—N2—N3—C2	0.1 (4)	C2—N1—C6—C11	158.4 (4)
C3—N2—N3—C2	−178.7 (3)	C1—N1—C6—C7	157.1 (3)
N3—N2—C1—N1	0.3 (4)	C2—N1—C6—C7	−20.4 (5)
C3—N2—C1—N1	178.9 (3)	C11—C6—C7—C8	−1.4 (5)
C2—N1—C1—N2	−0.5 (4)	N1—C6—C7—C8	177.3 (3)
C6—N1—C1—N2	−178.4 (3)	C6—C7—C8—C9	1.3 (6)
N2—N3—C2—N1	−0.4 (4)	C7—C8—C9—C10	0.2 (6)
C1—N1—C2—N3	0.6 (4)	C7—C8—C9—C12	−179.2 (4)
C6—N1—C2—N3	178.6 (3)	C8—C9—C10—C11	−1.6 (6)
C1—N2—C3—C4	1.5 (5)	C12—C9—C10—C11	177.9 (4)
N3—N2—C3—C4	−179.9 (3)	C7—C6—C11—C10	0.1 (5)
C1—N2—C3—C5	−122.8 (4)	N1—C6—C11—C10	−178.6 (3)
N3—N2—C3—C5	55.8 (4)	C9—C10—C11—C6	1.4 (6)
C1—N1—C6—C11	−24.1 (5)		

### (salt2) 1-Isopropyl-4-(4-methylphenyl)-1,2,4-triazol-1-ium iodide Hydrogen-bond geometry (Å, º)

**Table:**

*D*—H···*A*	*D*—H	H···*A*	*D*···*A*	*D*—H···*A*
C1—H1···I1^i^	0.95	3.06	3.901 (3)	149
C2—H2···I1^ii^	0.95	2.84	3.771 (4)	168
C3—H3···I1^iii^	1.00	3.00	3.870 (4)	146
C11—H11···I1^i^	0.95	2.98	3.930 (3)	174

Symmetry codes: (i) −*x*+1/2, *y*, *z*+1/2; (ii) −*x*+1, −*y*, −*z*+1; (iii) *x*, *y*, *z*+1.

### (salt3) 1-Isopropyl-4-phenyl-1,2,4-triazol-1-ium iodide Crystal data

**Table:**

C_11_H_14_N_3_^+^·I^−^	*F*(000) = 616
*M**_r_* = 315.15	*D*_x_ = 1.674 Mg m^−^^3^
Monoclinic, *P*2_1_/*n*	Mo *K*α radiation, λ = 0.71075 Å
*a* = 5.9326 (11) Å	Cell parameters from 11444 reflections
*b* = 17.826 (3) Å	θ = 3.4–27.6°
*c* = 12.129 (2) Å	µ = 2.54 mm^−^^1^
β = 102.897 (7)°	*T* = 173 K
*V* = 1250.3 (4) Å^3^	Prism, colorless
*Z* = 4	0.80 × 0.40 × 0.10 mm

### (salt3) 1-Isopropyl-4-phenyl-1,2,4-triazol-1-ium iodide Data collection

**Table:**

Rigaku XtaLAB mini diffractometer	2582 reflections with *I* > 2σ(*I*)
Detector resolution: 6.849 pixels mm^-1^	*R*_int_ = 0.044
ω scans	θ_max_ = 27.5°, θ_min_ = 3.5°
Absorption correction: multi-scan (*REQAB*; Rigaku, 1998)	*h* = −7→7
*T*_min_ = 0.356, *T*_max_ = 0.776	*k* = −23→23
12823 measured reflections	*l* = −15→15
2858 independent reflections	

### (salt3) 1-Isopropyl-4-phenyl-1,2,4-triazol-1-ium iodide Refinement

**Table:**

Refinement on *F*^2^	0 restraints
Least-squares matrix: full	Hydrogen site location: inferred from neighbouring sites
*R*[*F*^2^ > 2σ(*F*^2^)] = 0.023	H-atom parameters constrained
*wR*(*F*^2^) = 0.055	*w* = 1/[σ^2^(*F*_o_^2^) + (0.0167*P*)^2^ + 0.5394*P*] where *P* = (*F*_o_^2^ + 2*F*_c_^2^)/3
*S* = 1.09	(Δ/σ)_max_ = 0.002
2858 reflections	Δρ_max_ = 0.40 e Å^−^^3^
138 parameters	Δρ_min_ = −0.69 e Å^−^^3^

### (salt3) 1-Isopropyl-4-phenyl-1,2,4-triazol-1-ium iodide Special details

**Table:**

Geometry. All e.s.d.'s (except the e.s.d. in the dihedral angle between two l.s. planes) are estimated using the full covariance matrix. The cell e.s.d.'s are taken into account individually in the estimation of e.s.d.'s in distances, angles and torsion angles; correlations between e.s.d.'s in cell parameters are only used when they are defined by crystal symmetry. An approximate (isotropic) treatment of cell e.s.d.'s is used for estimating e.s.d.'s involving l.s. planes.

### (salt3) 1-Isopropyl-4-phenyl-1,2,4-triazol-1-ium iodide Fractional atomic coordinates and isotropic or equivalent isotropic displacement parameters (Å^2^)

**Table:**

	*x*	*y*	*z*	*U*_iso_*/*U*_eq_	
I1	0.15256 (2)	0.20965 (2)	0.66252 (2)	0.02352 (6)	
N1	0.3956 (3)	0.19026 (11)	0.39549 (15)	0.0187 (4)	
N2	0.5118 (3)	0.29644 (10)	0.46845 (17)	0.0209 (4)	
N3	0.3012 (3)	0.31018 (12)	0.39771 (16)	0.0236 (4)	
C1	0.5686 (4)	0.22566 (13)	0.46767 (19)	0.0212 (5)	
H1	0.7062	0.2033	0.5101	0.025*	
C2	0.2350 (4)	0.24481 (13)	0.35485 (19)	0.0226 (5)	
H2	0.0932	0.2359	0.3020	0.027*	
C3	0.6455 (4)	0.35740 (13)	0.53664 (19)	0.0257 (5)	
H3	0.7619	0.3342	0.5999	0.031*	
C4	0.7738 (5)	0.40192 (15)	0.4629 (2)	0.0392 (6)	
H4A	0.6625	0.4228	0.3982	0.047*	
H4B	0.8817	0.3688	0.4358	0.047*	
H4C	0.8599	0.4428	0.5073	0.047*	
C5	0.4824 (4)	0.40497 (14)	0.5871 (2)	0.0335 (6)	
H5A	0.4001	0.3730	0.6308	0.040*	
H5B	0.3705	0.4296	0.5262	0.040*	
H5C	0.5710	0.4430	0.6369	0.040*	
C6	0.3826 (4)	0.11147 (13)	0.36959 (18)	0.0205 (5)	
C7	0.5430 (4)	0.08035 (15)	0.3150 (2)	0.0294 (5)	
H7	0.6593	0.1105	0.2945	0.035*	
C8	0.5291 (5)	0.00422 (16)	0.2914 (2)	0.0393 (6)	
H8	0.6388	−0.0185	0.2556	0.047*	
C9	0.3563 (4)	−0.03878 (15)	0.3195 (2)	0.0376 (6)	
H9	0.3462	−0.0907	0.3016	0.045*	
C10	0.1980 (5)	−0.00676 (15)	0.3736 (2)	0.0349 (6)	
H10	0.0800	−0.0368	0.3928	0.042*	
C11	0.2112 (4)	0.06924 (14)	0.4001 (2)	0.0285 (5)	
H11	0.1047	0.0916	0.4382	0.034*	

### (salt3) 1-Isopropyl-4-phenyl-1,2,4-triazol-1-ium iodide Atomic displacement parameters (Å^2^)

**Table:**

	*U*^11^	*U*^22^	*U*^33^	*U*^12^	*U*^13^	*U*^23^
I1	0.01997 (9)	0.02622 (11)	0.02322 (10)	−0.00035 (5)	0.00235 (6)	−0.00040 (6)
N1	0.0184 (9)	0.0182 (9)	0.0187 (9)	0.0009 (7)	0.0021 (7)	−0.0004 (8)
N2	0.0204 (10)	0.0189 (10)	0.0220 (10)	0.0016 (7)	0.0015 (8)	0.0004 (8)
N3	0.0237 (10)	0.0198 (10)	0.0254 (10)	0.0050 (8)	0.0013 (8)	0.0028 (8)
C1	0.0205 (11)	0.0176 (11)	0.0235 (11)	0.0014 (9)	0.0009 (9)	−0.0013 (9)
C2	0.0206 (11)	0.0218 (13)	0.0236 (11)	0.0031 (9)	0.0011 (9)	0.0009 (10)
C3	0.0275 (12)	0.0162 (12)	0.0291 (12)	−0.0003 (9)	−0.0024 (9)	−0.0032 (10)
C4	0.0370 (14)	0.0268 (14)	0.0583 (18)	−0.0093 (11)	0.0202 (13)	−0.0114 (13)
C5	0.0430 (15)	0.0260 (14)	0.0333 (13)	−0.0031 (11)	0.0125 (11)	−0.0098 (11)
C6	0.0220 (10)	0.0183 (12)	0.0186 (10)	0.0023 (9)	−0.0008 (8)	0.0002 (9)
C7	0.0272 (12)	0.0267 (13)	0.0351 (13)	0.0014 (10)	0.0091 (10)	−0.0034 (11)
C8	0.0406 (15)	0.0309 (15)	0.0462 (16)	0.0097 (12)	0.0091 (12)	−0.0090 (13)
C9	0.0534 (17)	0.0160 (13)	0.0371 (14)	0.0016 (11)	−0.0032 (12)	−0.0042 (11)
C10	0.0454 (15)	0.0238 (14)	0.0340 (14)	−0.0097 (11)	0.0061 (11)	0.0029 (11)
C11	0.0350 (13)	0.0247 (13)	0.0271 (12)	−0.0025 (10)	0.0096 (10)	0.0004 (10)

### (salt3) 1-Isopropyl-4-phenyl-1,2,4-triazol-1-ium iodide Geometric parameters (Å, º)

**Table:**

N1—C1	1.348 (3)	C5—H5A	0.9800
N1—C2	1.374 (3)	C5—H5B	0.9800
N1—C6	1.437 (3)	C5—H5C	0.9800
N2—C1	1.306 (3)	C6—C11	1.380 (3)
N2—N3	1.371 (3)	C6—C7	1.390 (3)
N2—C3	1.484 (3)	C7—C8	1.386 (4)
N3—C2	1.301 (3)	C7—H7	0.9500
C1—H1	0.9500	C8—C9	1.382 (4)
C2—H2	0.9500	C8—H8	0.9500
C3—C5	1.515 (3)	C9—C10	1.384 (4)
C3—C4	1.520 (3)	C9—H9	0.9500
C3—H3	1.0000	C10—C11	1.390 (4)
C4—H4A	0.9800	C10—H10	0.9500
C4—H4B	0.9800	C11—H11	0.9500
C4—H4C	0.9800		
			
C1—N1—C2	105.57 (19)	C3—C5—H5A	109.5
C1—N1—C6	126.54 (19)	C3—C5—H5B	109.5
C2—N1—C6	127.88 (19)	H5A—C5—H5B	109.5
C1—N2—N3	111.62 (19)	C3—C5—H5C	109.5
C1—N2—C3	127.22 (19)	H5A—C5—H5C	109.5
N3—N2—C3	121.14 (18)	H5B—C5—H5C	109.5
C2—N3—N2	103.99 (18)	C11—C6—C7	122.2 (2)
N2—C1—N1	107.3 (2)	C11—C6—N1	118.8 (2)
N2—C1—H1	126.4	C7—C6—N1	119.0 (2)
N1—C1—H1	126.4	C8—C7—C6	118.3 (2)
N3—C2—N1	111.6 (2)	C8—C7—H7	120.9
N3—C2—H2	124.2	C6—C7—H7	120.9
N1—C2—H2	124.2	C9—C8—C7	120.3 (2)
N2—C3—C5	108.96 (19)	C9—C8—H8	119.8
N2—C3—C4	109.27 (19)	C7—C8—H8	119.8
C5—C3—C4	113.2 (2)	C8—C9—C10	120.5 (3)
N2—C3—H3	108.4	C8—C9—H9	119.7
C5—C3—H3	108.4	C10—C9—H9	119.7
C4—C3—H3	108.4	C9—C10—C11	120.1 (2)
C3—C4—H4A	109.5	C9—C10—H10	119.9
C3—C4—H4B	109.5	C11—C10—H10	119.9
H4A—C4—H4B	109.5	C6—C11—C10	118.5 (2)
C3—C4—H4C	109.5	C6—C11—H11	120.8
H4A—C4—H4C	109.5	C10—C11—H11	120.8
H4B—C4—H4C	109.5		
			
C1—N2—N3—C2	0.0 (2)	C1—N1—C6—C11	114.7 (3)
C3—N2—N3—C2	−178.7 (2)	C2—N1—C6—C11	−63.7 (3)
N3—N2—C1—N1	0.1 (3)	C1—N1—C6—C7	−65.1 (3)
C3—N2—C1—N1	178.8 (2)	C2—N1—C6—C7	116.5 (3)
C2—N1—C1—N2	−0.2 (2)	C11—C6—C7—C8	−0.3 (4)
C6—N1—C1—N2	−178.9 (2)	N1—C6—C7—C8	179.6 (2)
N2—N3—C2—N1	−0.1 (2)	C6—C7—C8—C9	1.3 (4)
C1—N1—C2—N3	0.2 (3)	C7—C8—C9—C10	−1.3 (4)
C6—N1—C2—N3	178.9 (2)	C8—C9—C10—C11	0.1 (4)
C1—N2—C3—C5	−135.4 (2)	C7—C6—C11—C10	−0.9 (4)
N3—N2—C3—C5	43.1 (3)	N1—C6—C11—C10	179.3 (2)
C1—N2—C3—C4	100.5 (3)	C9—C10—C11—C6	0.9 (4)
N3—N2—C3—C4	−81.0 (3)		

### (salt3) 1-Isopropyl-4-phenyl-1,2,4-triazol-1-ium iodide Hydrogen-bond geometry (Å, º)

**Table:**

*D*—H···*A*	*D*—H	H···*A*	*D*···*A*	*D*—H···*A*
C1—H1···I1^i^	0.95	2.87	3.744 (2)	153
C2—H2···I1^ii^	0.95	2.94	3.800 (2)	151
C3—H3···I1^i^	1.00	3.18	4.033 (2)	145

Symmetry codes: (i) *x*+1, *y*, *z*; (ii) *x*−1/2, −*y*+1/2, *z*−1/2.

### (salt4) 1-Methyl-4-phenyl-1,2,4-triazol-1-ium iodide Crystal data

**Table:**

C_9_H_10_N_3_^+^·I^−^	*F*(000) = 552
*M**_r_* = 287.10	*D*_x_ = 1.783 Mg m^−^^3^
Monoclinic, *C**c*	Mo *K*α radiation, λ = 0.71075 Å
*a* = 7.660 (2) Å	Cell parameters from 5405 reflections
*b* = 16.912 (5) Å	θ = 3.5–27.6°
*c* = 8.412 (3) Å	µ = 2.96 mm^−^^1^
β = 101.137 (7)°	*T* = 173 K
*V* = 1069.2 (6) Å^3^	Prism, colorless
*Z* = 4	0.31 × 0.23 × 0.13 mm

### (salt4) 1-Methyl-4-phenyl-1,2,4-triazol-1-ium iodide Data collection

**Table:**

Rigaku XtaLAB mini diffractometer	2359 reflections with *I* > 2σ(*I*)
Detector resolution: 6.849 pixels mm^-1^	*R*_int_ = 0.020
ω scans	θ_max_ = 27.5°, θ_min_ = 3.5°
Absorption correction: multi-scan (*REQAB*; Rigaku, 1998)	*h* = −9→9
*T*_min_ = 0.439, *T*_max_ = 0.681	*k* = −21→21
5472 measured reflections	*l* = −10→10
2420 independent reflections	

### (salt4) 1-Methyl-4-phenyl-1,2,4-triazol-1-ium iodide Refinement

**Table:**

Refinement on *F*^2^	Hydrogen site location: inferred from neighbouring sites
Least-squares matrix: full	H-atom parameters constrained
*R*[*F*^2^ > 2σ(*F*^2^)] = 0.018	*w* = 1/[σ^2^(*F*_o_^2^) + (0.0128*P*)^2^] where *P* = (*F*_o_^2^ + 2*F*_c_^2^)/3
*wR*(*F*^2^) = 0.037	(Δ/σ)_max_ < 0.001
*S* = 1.05	Δρ_max_ = 0.18 e Å^−^^3^
2420 reflections	Δρ_min_ = −0.36 e Å^−^^3^
119 parameters	Absolute structure: Flack *x* determined using 1112 quotients [(*I*^+^)-(*I*^-^)]/[(*I*^+^)+(*I*^-^)] (Parsons et al., 2013)
2 restraints	Absolute structure parameter: −0.012 (18)

### (salt4) 1-Methyl-4-phenyl-1,2,4-triazol-1-ium iodide Special details

**Table:**

Geometry. All e.s.d.'s (except the e.s.d. in the dihedral angle between two l.s. planes) are estimated using the full covariance matrix. The cell e.s.d.'s are taken into account individually in the estimation of e.s.d.'s in distances, angles and torsion angles; correlations between e.s.d.'s in cell parameters are only used when they are defined by crystal symmetry. An approximate (isotropic) treatment of cell e.s.d.'s is used for estimating e.s.d.'s involving l.s. planes.

### (salt4) 1-Methyl-4-phenyl-1,2,4-triazol-1-ium iodide Fractional atomic coordinates and isotropic or equivalent isotropic displacement parameters (Å^2^)

**Table:**

	*x*	*y*	*z*	*U*_iso_*/*U*_eq_	
I1	0.70097 (5)	0.18929 (2)	0.53544 (5)	0.03177 (8)	
N1	0.4911 (4)	0.06092 (17)	0.1783 (3)	0.0223 (6)	
N2	0.7314 (6)	0.0784 (2)	0.0734 (5)	0.0399 (12)	
N3	0.6399 (4)	0.14778 (18)	0.0768 (4)	0.0271 (7)	
C1	0.4981 (5)	0.1377 (2)	0.1394 (4)	0.0258 (8)	
H1	0.4152	0.1774	0.1546	0.031*	
C2	0.6381 (6)	0.0273 (2)	0.1362 (5)	0.0362 (10)	
H2	0.6682	−0.0270	0.1511	0.043*	
C3	0.7052 (12)	0.2216 (2)	0.0167 (8)	0.0414 (12)	
H3A	0.6248	0.2651	0.0304	0.050*	
H3B	0.7095	0.2158	−0.0984	0.050*	
H3C	0.8247	0.2332	0.0780	0.050*	
C4	0.3552 (5)	0.0234 (2)	0.2475 (5)	0.0227 (8)	
C5	0.2349 (7)	0.0696 (3)	0.3098 (6)	0.0333 (11)	
H5	0.2446	0.1256	0.3107	0.040*	
C6	0.0998 (6)	0.0327 (3)	0.3709 (6)	0.0378 (11)	
H6	0.0156	0.0636	0.4130	0.045*	
C7	0.0876 (7)	−0.0481 (3)	0.3707 (5)	0.0399 (12)	
H7	−0.0050	−0.0730	0.4128	0.048*	
C8	0.2093 (7)	−0.0936 (3)	0.3098 (5)	0.0381 (11)	
H8	0.1999	−0.1496	0.3106	0.046*	
C9	0.3449 (7)	−0.0583 (2)	0.2475 (5)	0.0296 (10)	
H9	0.4288	−0.0895	0.2057	0.035*	

### (salt4) 1-Methyl-4-phenyl-1,2,4-triazol-1-ium iodide Atomic displacement parameters (Å^2^)

**Table:**

	*U*^11^	*U*^22^	*U*^33^	*U*^12^	*U*^13^	*U*^23^
I1	0.03583 (13)	0.02441 (11)	0.03571 (12)	−0.00729 (17)	0.00855 (9)	−0.00034 (18)
N1	0.0210 (15)	0.0225 (15)	0.0248 (14)	0.0018 (13)	0.0077 (12)	0.0007 (12)
N2	0.035 (3)	0.0347 (19)	0.056 (3)	0.0041 (18)	0.024 (2)	0.0023 (16)
N3	0.0305 (17)	0.0241 (16)	0.0293 (16)	−0.0016 (14)	0.0120 (14)	0.0002 (13)
C1	0.0268 (19)	0.0248 (19)	0.0268 (18)	0.0041 (15)	0.0080 (16)	−0.0011 (15)
C2	0.033 (2)	0.025 (2)	0.056 (3)	0.0062 (17)	0.023 (2)	−0.0004 (18)
C3	0.055 (2)	0.0333 (19)	0.043 (3)	−0.014 (3)	0.026 (2)	−0.003 (3)
C4	0.0172 (18)	0.032 (2)	0.0181 (18)	0.0002 (17)	0.0023 (15)	0.0020 (16)
C5	0.031 (3)	0.036 (3)	0.034 (3)	0.001 (2)	0.009 (2)	−0.006 (2)
C6	0.025 (2)	0.058 (3)	0.033 (3)	−0.001 (2)	0.013 (2)	−0.005 (2)
C7	0.032 (3)	0.060 (3)	0.028 (2)	−0.014 (2)	0.004 (2)	0.005 (2)
C8	0.042 (3)	0.040 (3)	0.033 (2)	−0.012 (3)	0.009 (2)	0.005 (2)
C9	0.032 (3)	0.029 (2)	0.028 (2)	0.0018 (18)	0.0072 (19)	0.0048 (17)

### (salt4) 1-Methyl-4-phenyl-1,2,4-triazol-1-ium iodide Geometric parameters (Å, º)

**Table:**

N1—C1	1.343 (4)	C4—C9	1.384 (6)
N1—C2	1.368 (5)	C4—C5	1.386 (6)
N1—C4	1.435 (5)	C5—C6	1.389 (7)
N2—C2	1.297 (6)	C5—H5	0.9500
N2—N3	1.370 (5)	C6—C7	1.369 (7)
N3—C1	1.306 (5)	C6—H6	0.9500
N3—C3	1.471 (6)	C7—C8	1.381 (8)
C1—H1	0.9500	C7—H7	0.9500
C2—H2	0.9500	C8—C9	1.385 (7)
C3—H3A	0.9800	C8—H8	0.9500
C3—H3B	0.9800	C9—H9	0.9500
C3—H3C	0.9800		
			
C1—N1—C2	105.4 (3)	C9—C4—C5	121.3 (4)
C1—N1—C4	126.4 (3)	C9—C4—N1	119.2 (4)
C2—N1—C4	128.2 (3)	C5—C4—N1	119.4 (4)
C2—N2—N3	103.8 (4)	C4—C5—C6	119.0 (4)
C1—N3—N2	111.4 (3)	C4—C5—H5	120.5
C1—N3—C3	127.9 (4)	C6—C5—H5	120.5
N2—N3—C3	120.7 (4)	C7—C6—C5	120.2 (5)
N3—C1—N1	107.5 (3)	C7—C6—H6	119.9
N3—C1—H1	126.3	C5—C6—H6	119.9
N1—C1—H1	126.3	C6—C7—C8	120.4 (5)
N2—C2—N1	112.0 (3)	C6—C7—H7	119.8
N2—C2—H2	124.0	C8—C7—H7	119.8
N1—C2—H2	124.0	C7—C8—C9	120.6 (5)
N3—C3—H3A	109.5	C7—C8—H8	119.7
N3—C3—H3B	109.5	C9—C8—H8	119.7
H3A—C3—H3B	109.5	C4—C9—C8	118.5 (5)
N3—C3—H3C	109.5	C4—C9—H9	120.8
H3A—C3—H3C	109.5	C8—C9—H9	120.8
H3B—C3—H3C	109.5		
			
C2—N2—N3—C1	0.0 (4)	C1—N1—C4—C5	−12.9 (6)
C2—N2—N3—C3	179.3 (4)	C2—N1—C4—C5	167.9 (4)
N2—N3—C1—N1	−0.4 (4)	C9—C4—C5—C6	−1.0 (7)
C3—N3—C1—N1	−179.6 (4)	N1—C4—C5—C6	177.6 (4)
C2—N1—C1—N3	0.6 (4)	C4—C5—C6—C7	0.7 (7)
C4—N1—C1—N3	−178.7 (3)	C5—C6—C7—C8	0.0 (7)
N3—N2—C2—N1	0.4 (5)	C6—C7—C8—C9	−0.2 (7)
C1—N1—C2—N2	−0.6 (5)	C5—C4—C9—C8	0.7 (6)
C4—N1—C2—N2	178.6 (4)	N1—C4—C9—C8	−177.9 (4)
C1—N1—C4—C9	165.7 (4)	C7—C8—C9—C4	−0.1 (7)
C2—N1—C4—C9	−13.4 (6)		

### (salt4) 1-Methyl-4-phenyl-1,2,4-triazol-1-ium iodide Hydrogen-bond geometry (Å, º)

**Table:**

*D*—H···*A*	*D*—H	H···*A*	*D*···*A*	*D*—H···*A*
C1—H1···I1^i^	0.95	2.85	3.707 (4)	150
C2—H2···I1^ii^	0.95	2.94	3.811 (4)	153
C3—H3*B*···I1^iii^	0.98	3.10	4.079 (6)	176

Symmetry codes: (i) *x*−1/2, −*y*+1/2, *z*−1/2; (ii) *x*, −*y*, *z*−1/2; (iii) *x*, *y*, *z*−1.

### (salt5) 1-Benzyl-4-phenyl-1,2,4-triazol-1-ium bromide monohydrate Crystal data

**Table:**

C_15_H_14_N_3_^+^·Br^−^·H_2_O	*F*(000) = 1360
*M**_r_* = 334.22	*D*_x_ = 1.545 Mg m^−^^3^
Monoclinic, *C*2/*c*	Mo *K*α radiation, λ = 0.71075 Å
*a* = 24.783 (6) Å	Cell parameters from 10996 reflections
*b* = 8.996 (2) Å	θ = 3.2–27.7°
*c* = 13.089 (3) Å	µ = 2.86 mm^−^^1^
β = 100.068 (7)°	*T* = 173 K
*V* = 2873.3 (13) Å^3^	Prism, colorless
*Z* = 8	0.60 × 0.37 × 0.17 mm

### (salt5) 1-Benzyl-4-phenyl-1,2,4-triazol-1-ium bromide monohydrate Data collection

**Table:**

Rigaku XtaLAB mini diffractometer	2630 reflections with *I* > 2σ(*I*)
Detector resolution: 6.849 pixels mm^-1^	*R*_int_ = 0.031
ω scans	θ_max_ = 27.5°, θ_min_ = 3.2°
Absorption correction: multi-scan (*REQAB*; Rigaku, 1998)	*h* = −16→32
*T*_min_ = 0.321, *T*_max_ = 0.614	*k* = −11→9
6672 measured reflections	*l* = −16→17
3259 independent reflections	

### (salt5) 1-Benzyl-4-phenyl-1,2,4-triazol-1-ium bromide monohydrate Refinement

**Table:**

Refinement on *F*^2^	0 restraints
Least-squares matrix: full	Hydrogen site location: mixed
*R*[*F*^2^ > 2σ(*F*^2^)] = 0.043	H atoms treated by a mixture of independent and constrained refinement
*wR*(*F*^2^) = 0.098	*w* = 1/[σ^2^(*F*_o_^2^) + (0.0376*P*)^2^ + 4.9222*P*] where *P* = (*F*_o_^2^ + 2*F*_c_^2^)/3
*S* = 1.08	(Δ/σ)_max_ < 0.001
3259 reflections	Δρ_max_ = 0.75 e Å^−^^3^
189 parameters	Δρ_min_ = −0.63 e Å^−^^3^

### (salt5) 1-Benzyl-4-phenyl-1,2,4-triazol-1-ium bromide monohydrate Special details

**Table:**

Geometry. All e.s.d.'s (except the e.s.d. in the dihedral angle between two l.s. planes) are estimated using the full covariance matrix. The cell e.s.d.'s are taken into account individually in the estimation of e.s.d.'s in distances, angles and torsion angles; correlations between e.s.d.'s in cell parameters are only used when they are defined by crystal symmetry. An approximate (isotropic) treatment of cell e.s.d.'s is used for estimating e.s.d.'s involving l.s. planes.

### (salt5) 1-Benzyl-4-phenyl-1,2,4-triazol-1-ium bromide monohydrate Fractional atomic coordinates and isotropic or equivalent isotropic displacement parameters (Å^2^)

**Table:**

	*x*	*y*	*z*	*U*_iso_*/*U*_eq_	
Br1	0.91115 (2)	0.40151 (3)	0.12909 (3)	0.03409 (12)	
O1	0.95493 (13)	0.3722 (3)	0.3846 (2)	0.0497 (7)	
N1	0.93559 (9)	−0.0969 (2)	0.07656 (16)	0.0202 (5)	
N2	0.85547 (9)	−0.0072 (2)	0.02922 (17)	0.0230 (5)	
N3	0.84900 (10)	−0.1587 (3)	0.0213 (2)	0.0310 (6)	
C1	0.90660 (11)	0.0297 (3)	0.0619 (2)	0.0218 (6)	
H1	0.9206	0.1278	0.0731	0.026*	
C2	0.89834 (12)	−0.2097 (3)	0.0504 (2)	0.0282 (6)	
H2	0.9075	−0.3123	0.0533	0.034*	
C3	0.80795 (12)	0.0930 (3)	0.0032 (2)	0.0272 (6)	
H3A	0.7870	0.0662	−0.0657	0.033*	
H3B	0.8210	0.1966	−0.0005	0.033*	
C4	0.77094 (11)	0.0834 (3)	0.0831 (2)	0.0228 (6)	
C5	0.78422 (12)	0.1609 (3)	0.1760 (2)	0.0279 (6)	
H5	0.8160	0.2219	0.1883	0.034*	
C6	0.75109 (13)	0.1493 (3)	0.2505 (2)	0.0339 (7)	
H6	0.7605	0.2008	0.3145	0.041*	
C7	0.70429 (12)	0.0627 (3)	0.2321 (2)	0.0310 (7)	
H7	0.6817	0.0544	0.2834	0.037*	
C8	0.69055 (13)	−0.0116 (4)	0.1389 (3)	0.0357 (7)	
H8	0.6581	−0.0697	0.1257	0.043*	
C9	0.72382 (12)	−0.0019 (3)	0.0647 (2)	0.0306 (7)	
H9	0.7143	−0.0539	0.0009	0.037*	
C10	0.99369 (11)	−0.1085 (3)	0.11401 (19)	0.0219 (6)	
C11	1.02340 (12)	0.0196 (3)	0.1428 (2)	0.0248 (6)	
H11	1.0060	0.1140	0.1367	0.030*	
C12	1.07896 (12)	0.0078 (3)	0.1805 (2)	0.0289 (6)	
H12	1.0999	0.0947	0.2007	0.035*	
C13	1.10438 (13)	−0.1303 (4)	0.1889 (2)	0.0321 (7)	
H13	1.1425	−0.1380	0.2151	0.038*	
C14	1.07384 (13)	−0.2566 (4)	0.1590 (2)	0.0360 (7)	
H14	1.0912	−0.3511	0.1645	0.043*	
C15	1.01828 (12)	−0.2467 (3)	0.1210 (2)	0.0310 (7)	
H15	0.9974	−0.3334	0.1001	0.037*	
H1A	0.992 (2)	0.389 (5)	0.377 (4)	0.086 (17)*	
H1B	0.939 (3)	0.372 (7)	0.315 (5)	0.13 (3)*	

### (salt5) 1-Benzyl-4-phenyl-1,2,4-triazol-1-ium bromide monohydrate Atomic displacement parameters (Å^2^)

**Table:**

	*U*^11^	*U*^22^	*U*^33^	*U*^12^	*U*^13^	*U*^23^
Br1	0.0347 (2)	0.02343 (17)	0.0440 (2)	−0.00110 (13)	0.00651 (13)	−0.00295 (13)
O1	0.0535 (18)	0.0457 (15)	0.0542 (18)	0.0036 (13)	0.0215 (14)	0.0063 (13)
N1	0.0236 (12)	0.0192 (11)	0.0183 (10)	0.0007 (9)	0.0051 (9)	−0.0014 (9)
N2	0.0225 (13)	0.0221 (12)	0.0251 (12)	0.0027 (9)	0.0060 (10)	−0.0001 (9)
N3	0.0275 (14)	0.0210 (12)	0.0435 (15)	0.0013 (10)	0.0034 (11)	−0.0025 (11)
C1	0.0252 (15)	0.0185 (13)	0.0225 (13)	0.0019 (11)	0.0059 (11)	−0.0009 (10)
C2	0.0275 (16)	0.0201 (14)	0.0366 (16)	−0.0003 (12)	0.0044 (12)	−0.0026 (12)
C3	0.0237 (15)	0.0290 (15)	0.0287 (14)	0.0070 (12)	0.0039 (11)	0.0062 (12)
C4	0.0188 (14)	0.0224 (14)	0.0265 (14)	0.0070 (11)	0.0021 (11)	0.0042 (11)
C5	0.0215 (15)	0.0298 (15)	0.0316 (15)	−0.0024 (12)	0.0018 (12)	−0.0013 (12)
C6	0.0359 (18)	0.0369 (17)	0.0286 (15)	0.0007 (14)	0.0047 (13)	−0.0028 (13)
C7	0.0266 (16)	0.0319 (16)	0.0361 (17)	0.0041 (12)	0.0101 (13)	0.0057 (13)
C8	0.0233 (16)	0.0350 (18)	0.049 (2)	−0.0060 (13)	0.0068 (14)	0.0003 (15)
C9	0.0256 (16)	0.0309 (16)	0.0340 (16)	−0.0005 (12)	0.0014 (13)	−0.0059 (13)
C10	0.0235 (14)	0.0284 (14)	0.0144 (12)	0.0017 (11)	0.0050 (10)	0.0008 (10)
C11	0.0280 (16)	0.0260 (15)	0.0201 (13)	−0.0021 (12)	0.0036 (11)	0.0024 (11)
C12	0.0280 (16)	0.0380 (17)	0.0204 (13)	−0.0063 (13)	0.0039 (11)	0.0009 (12)
C13	0.0255 (16)	0.0476 (19)	0.0226 (14)	0.0063 (13)	0.0027 (12)	0.0032 (13)
C14	0.0331 (18)	0.0334 (17)	0.0403 (18)	0.0106 (14)	0.0027 (14)	0.0006 (14)
C15	0.0290 (17)	0.0265 (15)	0.0358 (16)	0.0054 (12)	0.0009 (13)	−0.0023 (13)

### (salt5) 1-Benzyl-4-phenyl-1,2,4-triazol-1-ium bromide monohydrate Geometric parameters (Å, º)

**Table:**

O1—H1A	0.95 (6)	C6—C7	1.382 (4)
O1—H1B	0.92 (7)	C6—H6	0.9500
N1—C1	1.342 (3)	C7—C8	1.381 (4)
N1—C2	1.375 (4)	C7—H7	0.9500
N1—C10	1.441 (4)	C8—C9	1.382 (4)
N2—C1	1.307 (3)	C8—H8	0.9500
N2—N3	1.374 (3)	C9—H9	0.9500
N2—C3	1.475 (3)	C10—C15	1.380 (4)
N3—C2	1.299 (4)	C10—C11	1.384 (4)
C1—H1	0.9500	C11—C12	1.383 (4)
C2—H2	0.9500	C11—H11	0.9500
C3—C4	1.508 (4)	C12—C13	1.388 (4)
C3—H3A	0.9900	C12—H12	0.9500
C3—H3B	0.9900	C13—C14	1.383 (5)
C4—C9	1.383 (4)	C13—H13	0.9500
C4—C5	1.391 (4)	C14—C15	1.383 (4)
C5—C6	1.384 (4)	C14—H14	0.9500
C5—H5	0.9500	C15—H15	0.9500
			
H1A—O1—H1B	98 (5)	C5—C6—H6	119.9
C1—N1—C2	105.8 (2)	C8—C7—C6	119.8 (3)
C1—N1—C10	126.0 (2)	C8—C7—H7	120.1
C2—N1—C10	128.2 (2)	C6—C7—H7	120.1
C1—N2—N3	111.7 (2)	C7—C8—C9	120.3 (3)
C1—N2—C3	127.5 (2)	C7—C8—H8	119.8
N3—N2—C3	120.7 (2)	C9—C8—H8	119.8
C2—N3—N2	103.7 (2)	C8—C9—C4	120.1 (3)
N2—C1—N1	107.2 (2)	C8—C9—H9	119.9
N2—C1—H1	126.4	C4—C9—H9	119.9
N1—C1—H1	126.4	C15—C10—C11	121.6 (3)
N3—C2—N1	111.7 (3)	C15—C10—N1	119.4 (2)
N3—C2—H2	124.2	C11—C10—N1	119.0 (2)
N1—C2—H2	124.2	C12—C11—C10	118.8 (3)
N2—C3—C4	111.3 (2)	C12—C11—H11	120.6
N2—C3—H3A	109.4	C10—C11—H11	120.6
C4—C3—H3A	109.4	C11—C12—C13	120.4 (3)
N2—C3—H3B	109.4	C11—C12—H12	119.8
C4—C3—H3B	109.4	C13—C12—H12	119.8
H3A—C3—H3B	108.0	C14—C13—C12	119.6 (3)
C9—C4—C5	119.6 (3)	C14—C13—H13	120.2
C9—C4—C3	120.6 (3)	C12—C13—H13	120.2
C5—C4—C3	119.8 (3)	C15—C14—C13	120.7 (3)
C6—C5—C4	120.0 (3)	C15—C14—H14	119.7
C6—C5—H5	120.0	C13—C14—H14	119.7
C4—C5—H5	120.0	C10—C15—C14	118.8 (3)
C7—C6—C5	120.2 (3)	C10—C15—H15	120.6
C7—C6—H6	119.9	C14—C15—H15	120.6
			
C1—N2—N3—C2	−0.1 (3)	C6—C7—C8—C9	1.1 (5)
C3—N2—N3—C2	−179.6 (2)	C7—C8—C9—C4	−0.5 (5)
N3—N2—C1—N1	0.0 (3)	C5—C4—C9—C8	−0.9 (4)
C3—N2—C1—N1	179.4 (2)	C3—C4—C9—C8	179.1 (3)
C2—N1—C1—N2	0.1 (3)	C1—N1—C10—C15	−177.3 (3)
C10—N1—C1—N2	−178.9 (2)	C2—N1—C10—C15	4.0 (4)
N2—N3—C2—N1	0.2 (3)	C1—N1—C10—C11	3.1 (4)
C1—N1—C2—N3	−0.1 (3)	C2—N1—C10—C11	−175.6 (3)
C10—N1—C2—N3	178.8 (2)	C15—C10—C11—C12	−0.7 (4)
C1—N2—C3—C4	−108.9 (3)	N1—C10—C11—C12	178.8 (2)
N3—N2—C3—C4	70.5 (3)	C10—C11—C12—C13	0.2 (4)
N2—C3—C4—C9	−99.5 (3)	C11—C12—C13—C14	0.3 (4)
N2—C3—C4—C5	80.5 (3)	C12—C13—C14—C15	−0.2 (5)
C9—C4—C5—C6	1.7 (4)	C11—C10—C15—C14	0.8 (4)
C3—C4—C5—C6	−178.3 (3)	N1—C10—C15—C14	−178.8 (2)
C4—C5—C6—C7	−1.1 (5)	C13—C14—C15—C10	−0.3 (5)
C5—C6—C7—C8	−0.3 (5)		

### (salt5) 1-Benzyl-4-phenyl-1,2,4-triazol-1-ium bromide monohydrate Hydrogen-bond geometry (Å, º)

**Table:**

*D*—H···*A*	*D*—H	H···*A*	*D*···*A*	*D*—H···*A*
C1—H1···Br1	0.95	2.59	3.455 (3)	151
C2—H2···Br1^i^	0.95	2.75	3.644 (3)	156
C11—H11···O1^ii^	0.95	2.55	3.247 (4)	130
O1—H1*A*···Br1^ii^	0.95 (6)	2.42 (6)	3.365 (3)	172 (4)
O1—H1*B*···Br1	0.92 (7)	2.43 (7)	3.341 (3)	170 (5)

Symmetry codes: (i) *x*, *y*−1, *z*; (ii) −*x*+2, *y*, −*z*+1/2.
